# Differential Analysis of Age, Gender, Race, Sentiment, and Emotion in Substance Use Discourse on Twitter During the COVID-19 Pandemic: A Natural Language Processing Approach

**DOI:** 10.2196/67333

**Published:** 2025-07-28

**Authors:** Julina Maharjan, Ruoming Jin, Jennifer King, Jianfeng Zhu, Deric Kenne

**Affiliations:** 1 Department of Computer Science Kent State University Kent, OH United States; 2 Department of Public Health Kent State University Kent, OH United States

**Keywords:** substance use, social media, deep learning, natural language processing, NLP, COVID-19, age, gender, race, sentiment, emotion, artificial intelligence, AI

## Abstract

**Background:**

User demographics are often hidden in social media data due to privacy concerns. However, demographic information on substance use (SU) can provide valuable insights, allowing public health policy makers to focus on specific cohorts and develop efficient prevention strategies, especially during global crises such as the COVID-19 pandemic.

**Objective:**

This study aimed to analyze SU trends at the user level across different demographic dimensions, such as age, gender, race, and ethnicity, with a focus on the COVID-19 pandemic. The study also establishes a baseline for SU trends using social media data.

**Methods:**

The study was conducted using large-scale English-language data from Twitter (now known as X) over a 3-year period (2019, 2020, and 2021), comprising 1.13 billion posts. Following preprocessing, the SU posts were identified using our custom-trained deep learning model (Robustly Optimized Bidirectional Encoder Representations From Transformers Pretraining Approach [RoBERTa]), which resulted in the identification of 9 million SU posts. Then, demographic attributes, such as user type, age, gender, race, and ethnicity, as well as sentiments and emotions associated with each post, were extracted via a collection of natural language processing modules. Finally, various qualitative analyses were performed to obtain insight into user behaviors based on demographics.

**Results:**

The highest level of user participation in SU discussions was observed in 2020, with a 22.18% increase compared to 2019 and a 25.24% increase compared to 2021. Throughout the study period, male users and teenagers increasingly dominated the SU discussions across all substance types. During the COVID-19 pandemic, user participation in prescription medication discussions was notably higher among female users compared to other substance types. In addition, alcohol use increased by 80% within 2 weeks after the global pandemic declaration in 2020.

**Conclusions:**

This study presents a large-scale, fine-grained analysis of SU on social media data, examining trends by age, gender, race, and ethnicity before, during, and after the COVID-19 pandemic. Our findings, contextualized with sociocultural and pandemic-specific factors, provide actionable insights for targeted public health interventions. This study establishes social media data (powered with artificial intelligence and natural language processing tools) as a valuable platform for real-time SU surveillance and prevention during crises.

## Introduction

### Overview

Substance use (SU) prevalence varies across demographics such as age, gender, race, and ethnicity. During the COVID-19 pandemic, these differences became more pronounced. The pandemic not only increased global SU, with overdose deaths rising by 29.4% [1], but also exacerbated societal and racial inequalities [2,3] and significantly impacted mental health [4-7]. As people often turn to substances as a coping mechanism during crises [8,9], the pandemic likely led to increased SU [10], particularly among populations considered vulnerable [11]. Investigating how these trends shifted across different demographic groups during the pandemic is crucial for understanding public health challenges and developing targeted interventions.

### Background

#### Gender, Age, and Racial Disparities in SU

According to the National Center for Drug Abuse Statistics (NCDAS) [12], men are more likely than women to use illicit drugs. In 2020, 22% of male individuals and 17% of female individuals used illegal drugs or misused prescription drugs within the last year, and the highest prevalence was among individuals aged 18 to 25 (39%), followed by those aged 26 to 29 (34%) [13]. Racial and ethnic disparities have always been prevalent in the history of drug use. For instance, White individuals were more likely to misuse prescription drugs, while other races were more likely to use other illicit drugs [14]. Similarly, opioid overdose death rates were higher in Black individuals [15]. Furthermore, the disparities by race and ethnicity were also found to be varied with age. For most SU disorders, estimated prevalence was higher for White participants at younger ages and Black participants at older ages [16].

#### Importance of Studying SU During the COVID-19 Pandemic

Given the preexisting disparities in SU, the COVID-19 pandemic likely exacerbated these trends. According to the Centers for Disease Control and Prevention [1], COVID-19 mortality rates from January 1, 2020, to May 31, 2024, varied significantly by age, gender, race, and ethnicity. Non-Hispanic White individuals accounted for 67% of deaths, individuals aged ≥75 years represented approximately 54% of deaths, and male individuals comprised 54% of the mortality rate. Simultaneously, the COVID-19 pandemic brought significant social and economic changes, disproportionately affecting minoritized populations and those considered underprivileged [16,17]. The rapid spread of the virus overwhelmed health care services, leading to lower priority for treatment for racial and ethnic minority people and individuals considered economically disadvantaged [18,19]. This discrimination exacerbated mental health issues [4], also highlighted by the US Centers for Disease Control and Prevention [1], which noted disparities in mental health and substance misuse among racial and ethnic minority populations due to unequal access to care, psychosocial stress, and social determinants of health. Given the disparities in COVID-19 mortality rates by age, gender, race, and ethnicity and the social and economic challenges exacerbated by the pandemic, studying SU trends across different demographic groups requires high attention. The disproportionate impact on minoritized populations and those considered underprivileged highlights the need to understand how these factors influenced SU, which will aid in developing targeted public health strategies to address the specific needs of populations considered affected.

#### Natural Language Processing and Its Application in Health Care

The advent of advanced natural language processing (NLP) techniques, particularly deep learning models, has revolutionized the health care field, enabling researchers to extract meaningful insights from vast and complex datasets for advanced decision-making. Health care data, which include unstructured sources such as medical reports, electronic health records, clinical trials, and social media, has traditionally posed significant challenges for analysts due to its volume, variability, and complexity. Recent advancements in NLP have addressed these challenges by facilitating tasks such as health information retrieval and extraction, text summarization, sentiment and emotion analysis, and the construction of medical ontologies and knowledge graphs. For instance, studies have demonstrated the utility of NLP in analyzing social media data to monitor public health trends, such as SU and mental health discussions during crises [20]. Similarly, NLP has been applied to identify patterns in clinical texts and patient narratives, enabling personalized health care interventions and improved decision-making [21]. These applications highlight the transformative potential of NLP in health care, particularly in leveraging unstructured data to address pressing public health challenges.

### Related Study

The study of SU prevalence across demographics has predominantly relied on survey-based research conducted by national agencies such as the Substance Abuse and Mental Health Services Administration (SAMHSA) [13] and the National Institute on Drug Abuse (NIDA) [22]. For example, the National Survey on Drug Use and Health (NSDUH) [12], administered by SAMHSA, provides comprehensive data on SU and mental health issues among the US population aged ≥12 years. Similarly, the Monitoring the Future [23] survey, funded by NIDA, focuses on SU patterns among youth by surveying middle and high school students (grades 8, 10, and 12). Both surveys provide detailed reports on the use of illicit and nonillicit drugs, disaggregated by age, gender, race, and ethnicity at a national level. In addition to these national surveys, various individual studies [16,24] have also explored SU disparities across demographics such as age, gender, race, and ethnicity.

While these surveys offer valuable insights, their scope is often limited by the diversity of true populations and the duration of the studied period. Traditional survey methods often rely on self-reported data, which can be affected by social desirability bias and recall errors. In addition, surveys are typically conducted annually or biennially, providing only periodic snapshots of SU trends. Moreover, the COVID-19 pandemic posed additional challenges for data collection; for example, SAMHSA 2020 was only able to collect data for the first and fourth quarters due to restrictions on in-person activities [13].

In contrast, social media data addresses many of these limitations. Social media platforms capture real-time, user-generated content that often reflects more authentic behaviors and sentiments. In addition to this, researchers have also shown the prevalence of SU discussions on social media [25-32], possibly due to its anonymity feature. Likewise, the continuous stream of data allows researchers [26,28,33-35] to monitor trends as they evolve, providing insights that are not possible with traditional survey methods. In addition, the vast amount of data available on social media enables a more detailed analysis across a large population [34], including those that might be underrepresented in surveys [32,36].

Despite the extensive research on SU trends, there remains a gap in understanding how these trends vary across different demographic groups, especially in the context of the COVID-19 pandemic. Existing studies have primarily relied on less diverse survey data or short-term real-time data, often overlooking the dynamic and nuanced shifts in SU behavior during global crises. This study aimed to address this gap by leveraging a large-scale social media dataset to provide a more granular and continuous analysis of SU trends across diverse demographics before, during, and after the pandemic. The following research questions (RQs) are designed to explore these trends in detail, offering insights into how age, gender, race, ethnicity, and emotional factors have influenced SU patterns during the study period:

1. What are the statistical distributions of overall SU posts and their users, categorized by key demographic variables in prepandemic, pandemic, and postpandemic periods?
2. To what extent do the SU patterns and demographic distributions observed in Twitter (now known as X) discourse from 2019 to 2021 correspond with or differ from the baseline trends reported by the NCDAS and other research?
3. What are the temporal trends in SU posts across different substance types throughout the study period, and how does the frequency of user posting behavior vary over time for each substance type?
4. How did the number of individuals discussing alcohol change within the first 2 weeks following the pandemic declaration compared to other users, and what short-term trends in user behavior emerged across different age groups, genders, races, and sentiments during this period?
5. What are the trends in user participation across different demographics for each substance type?
6. What emotional expressions are prevalent across all substance types?

## Methods

### Overview

**Figure 1:**
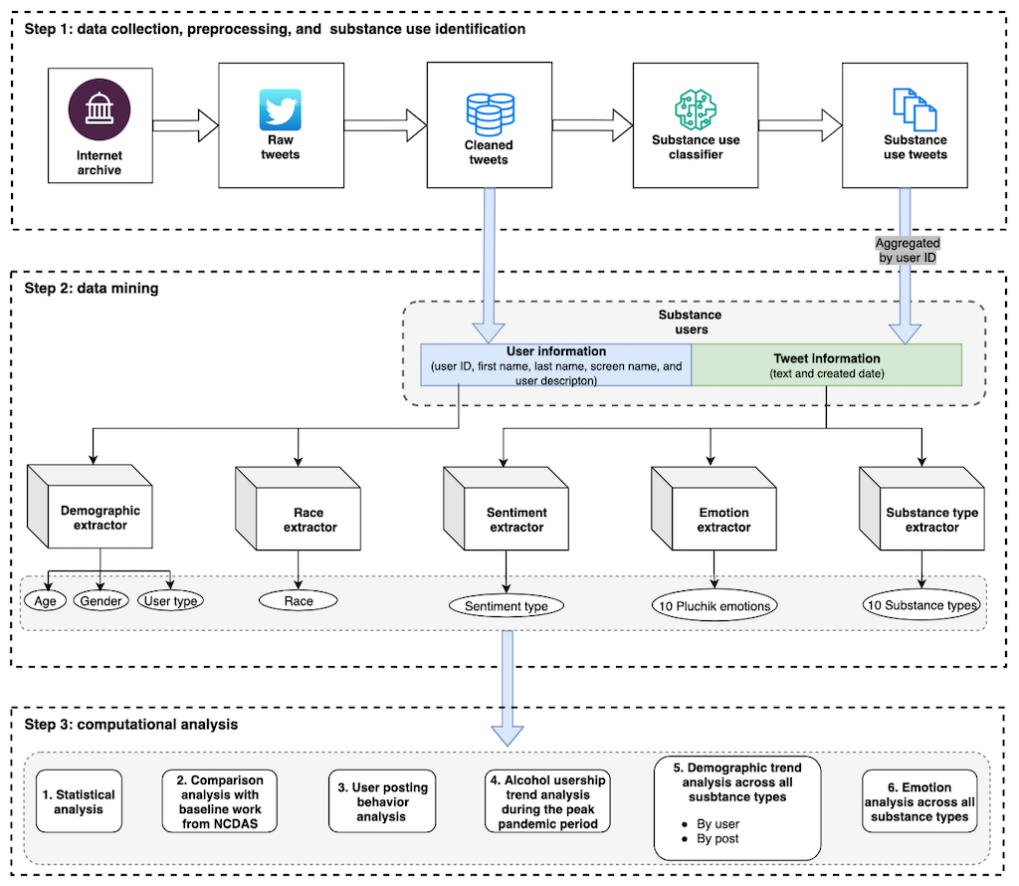
Methodology of the study design. NCDAS: National Center for Drug Abuse Statistics.

This study extends our previous research [37], which developed and validated a SU classifier using Robustly Optimized Bidirectional Encoder Representations From Transformers Pretraining Approach (RoBERTa) model [38] to identify SU posts from a cleaned dataset of 1.13 billion English-language posts (2019-2021). While our previous research [37] focused on analyzing SU at the post level, this study focuses on the user level. Thus, the key task of this study is to formulate the user base of SU posts from the previous study, followed by data mining (step 2) and computational analysis (step 3). As illustrated in Figure 1, step 1 encompasses the data collection, preprocessing, and SU identification modules. Step 2 involves user base formation and data mining methods for extracting demographic and emotional information associated with users and posts. Finally, step 3 involves computational analysis of the extracted data for users across various dimensions.

### Data Collection, Preprocessing, and SU Identification

We downloaded the raw tweet data from the Internet Archive [39], covering the period from January 2019 to December 2021. Unlike our previous study, which extracted only tweet information, this study also retrieved user information necessary for user-level analysis. Thus, the downloaded tweets had 2 types of information: tweet information and user information. The text information comprises text and the created date of a tweet, while user information comprises user ID, screen name, first name, last name, and user description.

The data processing of tweets was carried out in multiple steps. Initially, we filtered out all non-US tweets and duplicate or retweeted tweets to focus our research on English-language tweets and reduce redundancy, respectively. Then, we cleaned the text data by removing punctuation and stop words using the Natural Language Toolkit package and converted all characters to lowercase to maintain uniformity and prevent discrepancies caused by case sensitivity. Subsequently, we also replaced all the usernames, URLs, and hashtags in the post with the keywords *USER*, *HTTPURL*, and *HASHTAG* to hide the users’ identity and ease semantic understanding. Then, we performed lemmatization using the Natural Language Toolkit package to reduce words to their base form (eg, *drinking* to *drink*) to standardize text and improve consistency. Finally, we removed tweets containing <3 words, as these were deemed too brief to provide substantive insights. This comprehensive preprocessing approach resulted in a refined dataset of 1.13 billion cleaned tweets (3.1 million /1.13 billion, 26.84% in 2019; 4.5 million/1.13 billion, 40.05% in 2020; and 3.8 million/1.13 billion, 33.11% in 2021).

The cleaned tweets were then analyzed using a deep learning–based SU classifier, a pivotal element of our earlier research. This classifier was developed using a state-of-the-art model, RoBERTa [40], in conjunction with various techniques, such as transfer learning and a human-in-the-loop approach [41], to enhance its performance, achieving an accuracy rate of 80%. The successive validation on the sample data is also presented in the study by Maharjan et al [37]. The entire workflow for data collection, preprocessing, and SU identification is depicted in step 1 of Figure 1. This comprehensive phase concluded with 2.8 million, 3.5 million, and 2.5 million SU posts identified for the years 2019, 2020, and 2021, respectively, which we used in this study.

### Data Mining

#### Overview

Data mining constitutes a critical initial phase for conducting this research. At first, we formulated the SU dataset by users (or user base dataset) required in this work. Subsequently, we used this user base dataset to extract additional variables, where we used 5 different analytical modules: 2 focused on demographic variables (M3-Inference [35] and Ethicolr [40]) and 3 targeted at other relevant variables (Valence Aware Dictionary and Sentiment Reasoner [VADER] [42], SpanEmo [43], and a substance type extractor developed in our previous research [37]). Specifically, M3-Inference [44] and Ethicolr [40] were used to extract demographic information, such as age, gender, user type, race, and ethnicity from the user SU dataset. Alongside, VADER [42], SpanEmo [43], and the substance type extractor [37] were used to extract sentiment, emotional content, and substance type, respectively, from the post SU dataset. In the following sections, we provide detailed descriptions of the user base dataset and each of the 5 extraction modules.

#### SU User Base Dataset

For this study, we considered unique users who had posted at least 1 SU-related post. If the user has never posted any SU-related posts, their posts would never be included in SU posts, and the user would not be considered a substance user. We used a metadata field called *user ID* which served as a unique identifier for all users on the Twitter platform. Technically, we used identified SU posts from step 1 and aggregated them by unique user IDs to obtain the unique user base for this study, as shown in Figure 1. For example, if we had 10 SU posts, where 3 posts belonged to user A, 2 posts to user B, and 5 posts to 5 distinct users (C, D, E, F, and G), then we aggregated these posts by unique user IDs, such that there would be 7 unique SU users—A, B, C, D, E, F, and G. This step resulted in user base datasets of 2,131,457 for 2019, 2,604,123 for 2020, and 2,553,235 for 2021. After the user dataset was formulated, we further retrieved the user metadata for each user by performing a joint operation on the aggregated user base dataset and the original cleaned dataset. Thus, the final dataset used in this study included both tweet and user information. Tweet data included the content and creation date, while user data comprised user ID, first name, last name, screen name, and other profile details. This information served as an input for successive modules to extract additional variables such as age, gender, race, sentiment, and emotion.

#### Demographic (Age, Gender, and User Type) Extraction Using M3-Inference

In this study, we implemented the M3-Inference model [35] to extract demographic information, specifically age group, gender, and user type, from Twitter accounts. M3-Inference is an open-source Python implementation of a multimodal deep learning system, trained on extensive datasets, including Twitter, IMDB, and Wikipedia. The model’s architecture enables it to simultaneously predict 3 key demographic attributes: multimodal capabilities, which allow processing of both image and text features (we only used text features to perform our work); multilingual support, which includes 32 languages; and multiattribute prediction, which facilitates simultaneous forecasting of age, gender, and user type.

In terms of classification, the model treats gender (female or male) and user type (human or organization) as binary classification tasks, while age is categorized into 4 distinct groups: ≤18, 19 to 29, 30 to 39, and ≥40 years. For our analysis, we used a text-only pipeline to derive demographic predictions. This pipeline involved generating character-based embeddings for each textual input (username, screen name, and biography) and passing them through a 2-layer bidirectional character-level long short-term memory network.

To validate the M3-Inference model’s efficacy in predicting demographic attributes, we collected profile information from 50 known Twitter users (as detailed in Table S1 in Multimedia Appendix 1). For the age classification, we combined the 19 to 29, 30 to 39, and ≥40 years age groups into a single nonteenager category, thereby reformulating the age prediction as a binary classification task. The model’s performance metrics on the collected validation data indicated an accuracy of 99.05% for user type, 95% for gender, and 89% for age classification, with corresponding *F*_1_-scores of 0.98, 0.94, and 0.73, respectively.

#### Race and Ethnicity Extraction Using Ethnicolr

To infer the racial and ethnic backgrounds of individuals from their names, we used the Ethnicolr Python library [40]. This tool leverages several models based on different datasets, including US census data, Wikipedia entries, and Florida voter registration records, to predict the likelihood of an individual’s race and ethnicity. The model has 3 models depending on the type of dataset it is trained on. In our case, we used the Census Last Name Model, which was trained on US census data [45] from the years 2000 and 2010. This model estimates the percentage likelihood that an individual belongs to 4 main racial and ethnic categories, such as White, Black, Asian or Pacific Islander, or Hispanic. The predictions are appended as additional columns in the dataset, providing a probabilistic breakdown of racial composition. We verified the model on our sample data, where the model achieved an accuracy of 90%. The sample-predicted data are presented in Table S2 in Multimedia Appendix 1. However, in our study, we were able to extract the race information for only those posts that had the first name and last name present in the tweets; otherwise, the identification was not accomplished. Hence, approximately 65% (4.3 million/6.6 million) of the total users were identified, as detailed in Table S6 in Multimedia Appendix 1.

#### Sentiment Extraction Using VADER

VADER [42] is an open-source sentiment analysis tool designed specifically for analyzing social media text. It combines a lexicon-based approach with contextual rules to determine the sentiment of text as positive, negative, or neutral. VADER’s lexicon assigns sentiment scores to words on a scale from −4 (very negative) to +4 (very positive). Contextual adjustments are made through several mechanisms; punctuation, such as exclamation points, can amplify sentiments; capitalization highlights intensity, with all-caps being more emphatic; degree modifiers, such as “very,” strengthen sentiment; and conjunctions, such as “but,” can alter sentiment direction. The tool calculates a compound score, ranging from −1 (very negative) to +1 (very positive), by summing these adjusted scores. This method enables VADER to effectively capture both explicit and nuanced emotional expressions, providing a quick and reliable measure of overall sentiment in large volumes of text. The predicted sentiments for sample tweets are presented in Table S3 in Multimedia Appendix 1.

#### Emotion Extraction Using SpanEmo

SpanEmo [43] is a deep learning–based multilabel emotion recognition model. It analyzes text segments (spans) and classifies each span according to the emotions it conveys. The keywords associated with each emotion class are presented in Table S4 in Multimedia Appendix 1. This is particularly useful in complex texts where different parts may express different emotions. The tool uses NLP techniques to understand the context and semantic meanings of words and phrases, which allows it to accurately detect emotions even in nuanced or mixed emotional content. The model is based on bidirectional encoder representations from transformers, which takes the number of emotion classes (|C|=10) and a sequence “s” as inputs formatted with standard tokens (start_of_token [CLS] and separator_token [SEP]) as [CLS] + [C] + [SEP] + s. The encoding of emotion classed in the input makes the model learn the association between the emotion classes and the words in the input sentence, which is why it outperforms existing emotion classifiers. The model outputs 10 multiemotion classes, namely, anger, anticipation, disgust, fear, joy, love, optimism, pessimism, sadness, surprise, and trust. Before using this module, we finetuned this model on the SemEval-2018 multilabel emotion classification dataset [45] and achieved a *F*_1_-micro score of 0.70. The predicted emotions for sample tweets are presented in Table S5 in Multimedia Appendix 1.

#### Substance Type Identification

The substance type identification module is also based on our previous research [37]. In previous research [37], we considered the 10 primary substance types categorized together based on their pharmacological and behavioral effects and used the list of keywords from NIDA [22] to formulate keyword-based identification. The 10 types of substances were tobacco, alcohol, cannabinoids, opioids, stimulants, club drugs, hallucinogens, dissociative drugs, prescription medications, and other compounds.

### Theme Identification

In this study, we used 6 key themes that we formulated in our previous study [37]. The 6 key themes were COVID-19, economic factors, social influences, mental health, supply chain disruptions, and health care disruptions. The themes were formulated using latent Dirichlet allocation token analysis and were based on significant COVID-19 factors, such as stress and concerns related to COVID-19, economic instability, social dynamics, mental health issues, and disruptions in drug supply and health care services.

### Computational Analysis

In this study, we used 2 primary statistical techniques: trend and comparison analysis, along with sentiment and emotion analysis.

#### Trend and Comparison Analysis

To explore temporal patterns in SU discussions, we conducted a trend analysis, examining the frequency of posts over time. This allowed us to compare SU trends before, during, and after the pandemic. We further performed comparative analysis to assess differences in SU discussions across demographic categories, including age, gender, and race, identifying key disparities and dominant trends.

#### Sentiment and Emotion Analysis

We applied the VADER model to perform sentiment analysis, classifying the overall tone of posts (positive, negative, or neutral) related to SU. In addition, the SpanEmo model was used for emotion detection, allowing us to identify and categorize emotional expressions (eg, joy, anger, and sadness) linked to specific substances.

These methods provided insight into both the temporal dynamics of SU discussions and the emotional context in which they occurred.

### Ethical Considerations

To ensure the privacy and confidentiality of individuals whose data were analyzed, all study data underwent a rigorous deidentification process before analysis. The data for this study were sourced from publicly available platforms [39], containing no identifiable personal information. In addition, the sample posts were preprocessed to transform them into tokens, effectively obscuring any details that could reveal users’ identities. Our research was supported by the SAMHSA Strategic Prevention Framework-19 (grant 6H79SP081502), which was approved by the institutional review board at Kent State University (IRB20-182).

## Results

### Overview

In this study, we present a fine-grained demographic analysis of SU discourse on Twitter from a dual perspective: by post and by user. After preprocessing and identifying SU-related content, our final dataset included 2,799,726; 3,502,171; and 2,553,235 posts and 2,131,457; 2,604,123; and 1,946,742 users in 2019, 2020, and 2021, respectively. In the following sections, we first present a substantial summary of SU trends across all demographic dimensions: user type, gender, age group, race and ethnicity, and sentiment. Then, we compare our results with survey-based baseline research from NCDAS. Furthermore, we analyze the user trends on different substances, where alcohol users were found to be the prime users during the peak pandemic (March 2020 to June 2020) period. Hence, we performed a detailed analysis of alcohol users and posts during this time. In addition to this, we present the trends of all SU by users across 5 dimensions for each substance type. Finally, we present radar plots to understand the associated emotions with each substance type.

### RQ 1: What Are the Statistical Distributions of Overall SU Posts and Their Users, Categorized by Key Demographic Variables in Prepandemic, Pandemic, and Postpandemic Periods?

**Figure 2:**
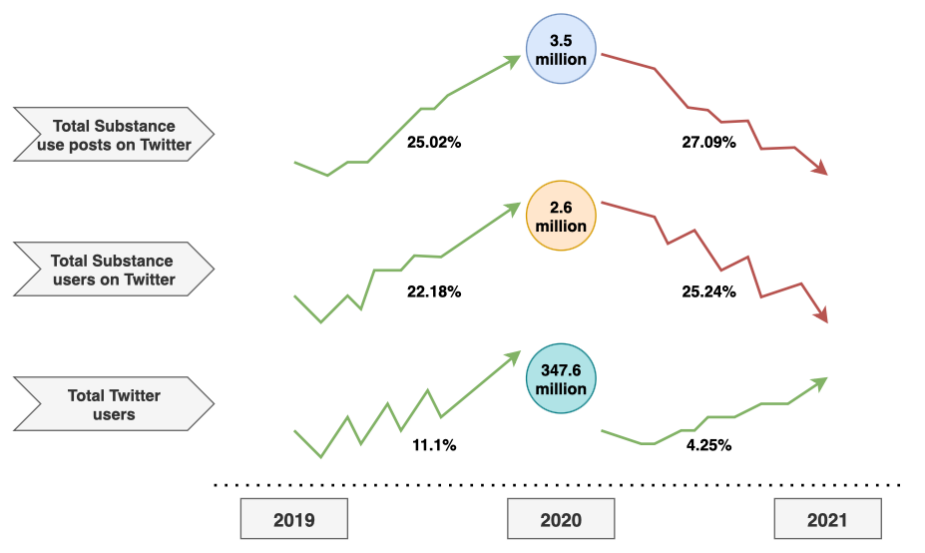
Overview of trends among Twitter users, substance users, and related posts.

Our key findings from the statistical analysis are presented in Table S6 in Multimedia Appendix 1, which summarizes the distribution of identified SU posts by both posts and users, further segmented by various categories, including user type, gender, age group, sentiment, race, and ethnicity. The Twitter user base has been expanding annually, with increases of 11.1% in 2020 and 4.25% in 2021 [46]. This growth is contextualized in Figure 2, where we illustrate the trends in the Twitter user base and SU in 2020, comparing both posts and users to prepandemic and postpandemic years. Notably, despite the increase in Twitter users, the marginal decline in SU posts and users in 2021 implies that SU was significantly higher in 2020.

**Figure 3:**
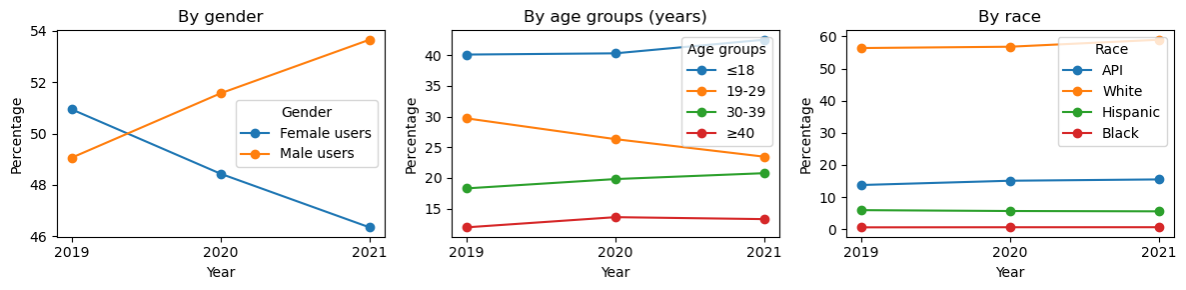
Trends in substance use by gender, age group, race, and ethnicity. API: Asian or Pacific islander.

Figure 3 presents the line plots depicting SU among the Twitter user base, categorized by gender, age group, race, and ethnicity. Statistics from Twitter users by gender [46] revealed that male users consistently outnumber female users on Twitter, with a distribution of 68% male users and 32% female users in 2020. In contrast, our analysis indicates that among substance users in 2020, 52% were male users and 48% were female users. This suggests that, despite a smaller female user base, female substance users represent a significant proportion of the overall female demographic on Twitter. As shown in Figure 3 (by gender), SU among female users increased from 2019 to 2021, whereas male users showed a declining trend over the same period.

**Figure 4:**
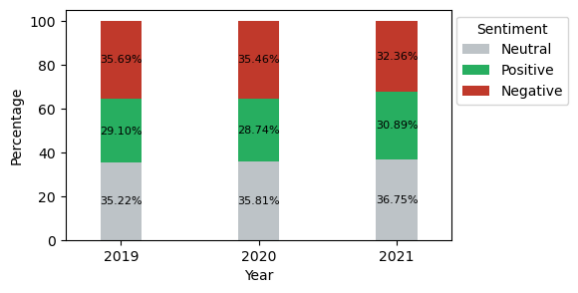
Sentiment distribution in substance use posts from 2019 to 2021.

Similarly, according to statistics from Twitter users [46], the highest levels of user engagement are found in the age group of 18 to 35 years. Moreover, our analysis also revealed a similar trend in substance users, as shown in Figure 3 (by age group), where a greater number of younger users were identified as substance users. Notably, our analysis indicated an increasing trend in SU among teenagers (≤18 years), alongside a decline among individuals aged 19 to 29 years. Our analysis identified race and ethnicity for 64.71% (1,811,516/2,131,457) of users in 2019, 64.99% (2,275,943/2,604,123) in 2020, and 66.51% (1,723,470/1,946,742) in 2021, representing approximately two-thirds of the total user base. Among these identified users, White individuals were the most prevalent across all years, as shown in Figure 3 (by race and ethnicity). The potential reasons and mitigation techniques are further discussed in the Limitations section. In addition to this, the sentiment distribution of SU posts revealed that the posts during 2019 and 2020 were highly associated with negative comments compared to 2021, as shown in Figure 4. Nevertheless, the increasing trend in positive and neutral comments after the pandemic suggests that 2020 was marked by relatively higher negative influences on SU.

### RQ 2: To What Extent Do the SU Patterns and Demographic Distributions Observed in Twitter (Now Known as X) Discourse From 2019 to 2021 Correspond With or Differ From the Baseline Trends Reported by the NCDAS and Other Research?

#### Overview

To evaluate the extent to which the SU patterns and demographics observed in Twitter discourse align with or deviate from baseline trends reported by NCDAS, we conducted a comparative analysis of both datasets. The NCDAS provides comprehensive annual reports on SU across various demographics, which serve as a benchmark for understanding broader trends. Our analysis focuses on comparing these established trends with the data extracted from Twitter posts spanning 2019 to 2021. This comparison aims to identify consistencies or discrepancies in SU trends and demographic patterns between the 2 sources.

#### Key Findings From Twitter Discourse

**Figure 5:**
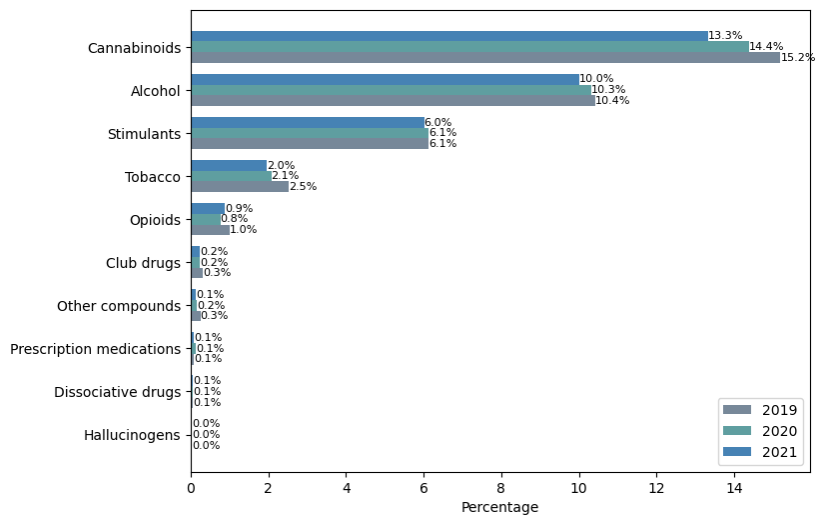
Trends in substance use discourse on Twitter in 2019, 2020, and 2021.

**Figure 6:**
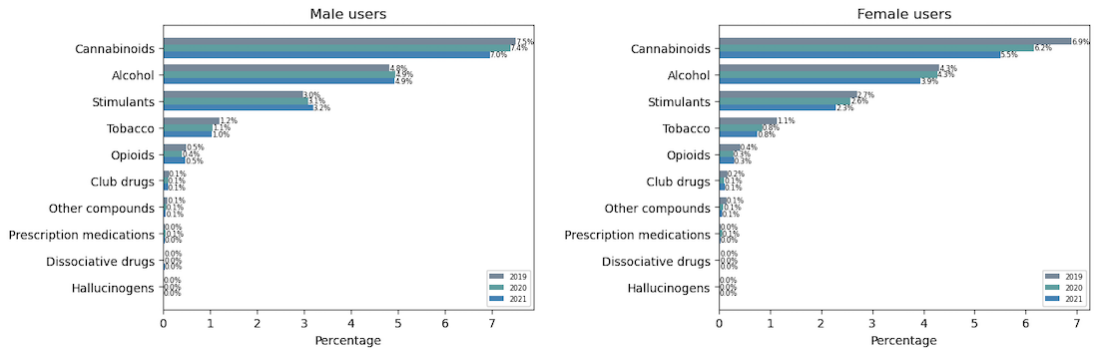
Gender-based trends in substance use discourse on Twitter in 2019, 2020, and 2021.

**Figure 7:**
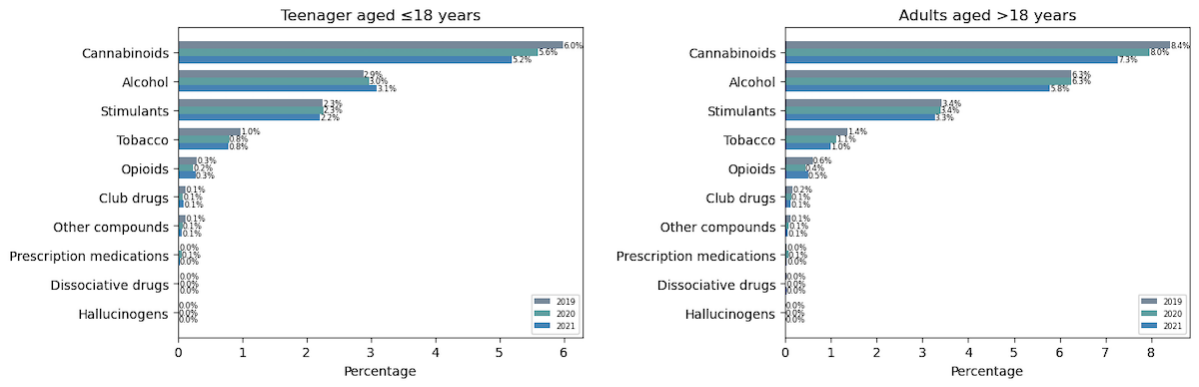
Age-based trends in substance use discourse on Twitter in 2019, 2020, and 2021.

The user distribution identified substance types and highlighted cannabinoids, stimulants, and opioids as the top 3 illicit substances discussed on Twitter (Figure 5). Demographically, male users dominated all substance types in all studied periods (Figure 6). Across the age group, SU was observed to be highest in teenagers aged ≤18 years (Figure 7). User participation in cannabinoid discussions remained the highest among all substance types, though it showed a declining trend among both adults and teenagers. Teenagers (aged ≤18 years) showed declines of 0.29% and 0.07% from 2019 to 2020 and 2020 to 2021, respectively, while adults (aged >18 years) showed declines of 0.52% and 0.07% over the same periods. For both opioids and stimulants, adults aged ≥40 years were observed to be highly involved among all age groups in all studied periods. Teenagers (aged ≤18 years) showed a decline in opioid use from 2019 to 2021. Similarly, the effect of the COVID-19 lockdown was evidenced in alcohol users profoundly (also supported by biweekly distribution charts in RQ 3; Figure 9), which increased by 80% in just 2 weeks after the global pandemic was declared on March 15, 2020.

#### Comparison With NCDAS Trends

Both the reports from NCDAS and our analysis have highlighted cannabinoids and stimulants as the top 2 illicit drugs in the study period. While these alignments suggest convergent validity, we note that Twitter discourse reflects both personal experiences and public commentary, whereas NCDAS measures self-reported use through standardized surveys. Although opioid-related usership on Twitter did not rank among the top substance discussions, as reported in NCDAS [12], both our study and the SAMHSA 2020 report [13] showed a declining trend. From 2019 to 2020, opioid mentions declined by 8.1% in the SAMHSA report and by 25% in our study. This difference in magnitude may reflect Twitter’s real-time sensitivity to news events versus surveys’ annualized behavioral data. Our result shows that opioid use was mostly prevalent in adults (aged >30 years) compared to teenagers (aged <18 years). This is likely supported by the overdose deaths report [22], where 75% of overdose deaths in adults were from opioids. Likewise, club drugs are widely known to be more commonly used by young people in higher-income settings. The same can be seen in this study, where teenagers were observed to be highly involved compared to other age groups. A similar trend of SU in terms of gender was observed in both studies. Male users were actively involved in all substance types, except for a few. The exception was prescription medication, which showed a higher prevalence among female users in both studies as shown in Table 1.

**Table 1:** User demographics in 2020.

Drug	By gender, (n=2,604,123), n (%)		By age group (y), (n=2,604,123), n (%)	
	Female users	Male users	≤18 years	>18 years
Cannabinoids	160,668 (6.17)	192,436 (7.39)	145,801 (5.6)	207,303 (7.96)
Alcohol	111,308 (4.27)	128,797 (4.95)	77,319 (2.97)	162,786 (6.25)
Stimulants	66,939 (2.57)	80,283 (3.08)	58,848 (2.26)	88,374 (3.39)
Tobacco	22,044 (0.85)	27,554 (1.06)	20,619 (0.79)	28,979 (1.11)
Opioids	7315 (0.28)	10,441 (0.4)	6235 (0.24)	11,521 (0.44)
Club drugs	2785 (0.11)	2805 (0.11)	1952 (0.07)	3638 (0.14)
Other compounds	2227 (0.09)	1900 (0.07)	1885 (0.07)	2242 (0.09)
Prescription medications	1751 (0.07)	1649 (0.06)	1378 (0.05)	2022 (0.08)
Dissociative drugs	618 (0.02)	657 (0.03)	480 (0.02)	795 (0.03)
Hallucinogens	260 (0.01)	348 (0.01)	252 (0.01)	356 (0.01)

#### Trend in Alcohol Users in 2020 From Other Survey-Based Research

The rise in alcohol use observed in our analysis during the peak pandemic period is supported by multiple studies [45,47]. Notably, our social media data detected this surge within weeks, while surveys [45,47] reported it months later, highlighting Twitter’s value for rapid monitoring, though with different population biases. A study by the United States Census Bureau [45] reported that alcohol consumption was observed to be highest as soon as college was closed during the pandemic lockdown. The study found that alcohol use was high for the users with mental health issues and low for those who received social support during the peak time; however, these patterns did not persist over time. A similar result was observed from our theme analysis detailed in RQ 4, where both social and mental health themes were observed as highly associated with alcohol posts during the peak pandemic period, March 15, 2020, to March 31, 2020. Likewise, a study by Lechner et al [48] also demonstrated that alcohol consumption was high during the peak pandemic period, which they associated with COVID-19–related stress, followed by availability of alcohol and boredom.

### RQ 3: What Are the Temporal Trends in SU Posts Across Different Substance Types Throughout the Study Period, and How Does the Frequency of User Posting Behavior Vary Over Time for Each Substance Type?

**Figure 8:**
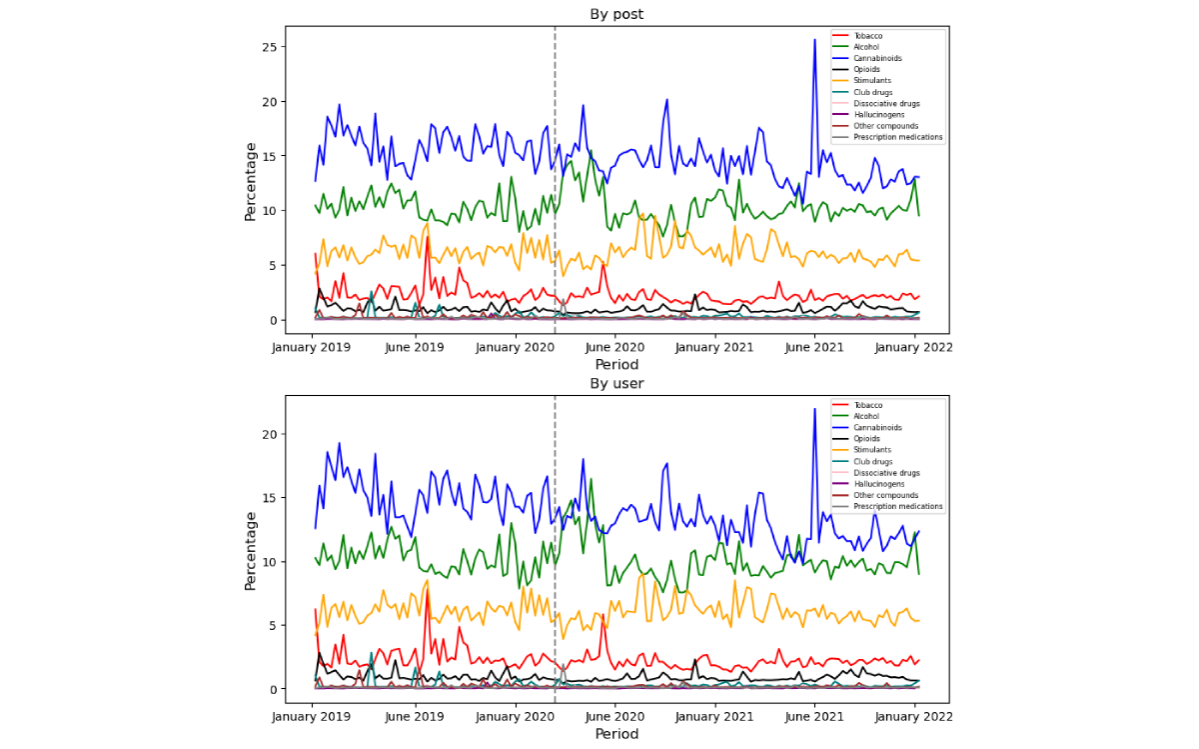
Weekly distribution across all substance types at the post and user level.

The temporal trend analysis gives the nuance of change of proportions with respect to time. In our study, we plotted weekly trends for all substance types for both users and posts (Figure 8). First, we identified substance type for each post using our keyword-based methods, as detailed in our previous research [37]. Moreover, to further analyze user posting behavior, we aggregated posts by unique users. Our analysis covers the period from 2019 to 2021 and presents data on a weekly basis, capturing both short-term fluctuations and long-term trends. The plot highlights cannabinoids as the most constantly discussed substance among all, followed by alcohol, stimulants, tobacco, and others. While the cannabinoid posts were the most frequent across all study periods, the alcohol users’ proportion increased sharply after the pandemic declaration day (March 15, 2020; as shown after the dotted gray line in Figure 8), demanding a detailed focus. Therefore, we present a detailed analysis of alcohol users during this period in our next question.

### RQ 4: How Did the Number of Individuals Discussing Alcohol Change Within the First 2 Weeks Following the Pandemic Declaration Compared to Other Users, and What Short-Term Trends in User Behavior Emerged Across Different Age Groups, Genders, Races, and Sentiments During This Period?

#### Trend Analysis on Alcohol Users During the Peak Pandemic Period

Our weekly trend analysis from RQ 3 highlighted that after the pandemic declaration, alcohol users surpassed all other substance users, including cannabinoid users (which was the highest discussed substance throughout the study period). Hence, we drill down on the alcohol users to understand if the increase in trend is associated with COVID-19.

**Figure 9:**
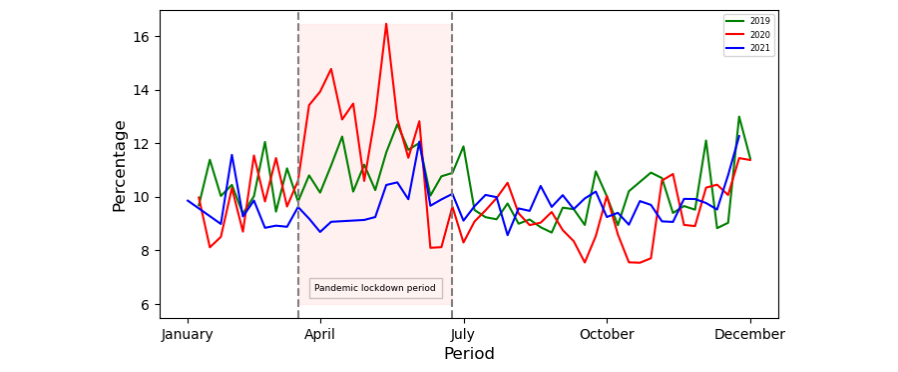
Alcohol: weekly user distribution in 2019, 2020, and 2021.

First, we compared weekly user trends during the pandemic year with the preceding year (2019) and following year (2021), as shown in Figure 9. The highlighted period from March 15 to June 15 marks the “pandemic lockdown period.” The visualization shows a dramatic increase in alcohol-related users during the pandemic year, while proportions in other periods remained consistent with the preceding and following years.

#### Demographic Trends on Alcohol Users During the Peak Pandemic Period

**Figure 10:**
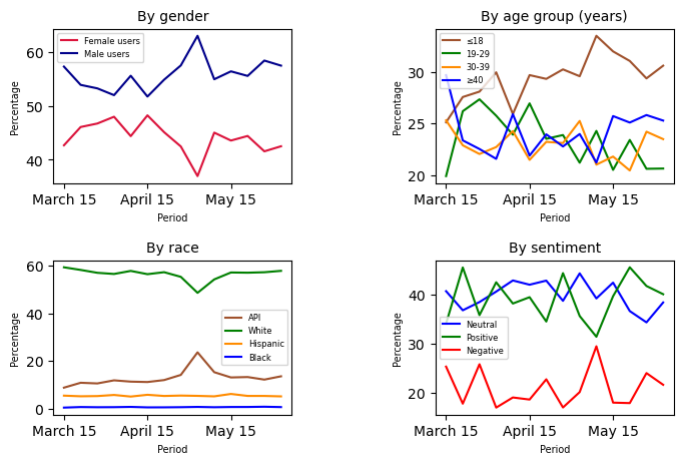
User distribution during the peak pandemic lockdown period (March 15, 2020, to June 15, 2020). API: Asian or Pacific islander; neg: negative; neu: neutral; pos: positive.

To further analyze patterns among alcohol users, we examined the pandemic lockdown period (from March 15, 2020, to June 15, 2020) in a weekly manner, segmented by age group, gender, race, and sentiment, as shown in Figure 10. Each subplot allows a comparative analysis within these specific demographic or sentiment groups. The gender and age analysis during this period show that male users and teenagers aged ≤18 years) were more involved in alcohol discussions compared to female users and other age groups, respectively. Likewise, increasing trends were observed among male users and teenagers, as well as among White users in the race analysis. However, the sentiments during this period were mostly neutral and positive.

#### Posts’ Content (Theme and Topic) Analysis on Alcohol Use During the Peak Pandemic Period

We performed a detailed analysis on the content of posts, where we derived the underlying themes (COVID-19, economic, social, mental health, supply disruption, and medical disruption) associated with the posts using a keyword method from our previous research [37]. The weekly distribution of alcohol posts in each theme is presented in Table 2. The distribution showed that alcohol-related discussions on peaked during the second week of March (March 15, 2020) across all themes, except the economic theme. Further analysis showed a significant increase in alcohol-related discussions during the week of March 15, 2020, particularly within the themes of social impact, mental health, supply disruption, and medical disruption.

**Table 2:** Weekly distribution of alcohol-related posts across all themes from February 2020 to May 2020.

Week	COVID-19, n (%)	Economic, n (%)	Social, n (%)	Mental health, n (%)	Supply distribution, n (%)	Medical disruption, n (%)
February 1, 2020 (n=6635)	95 (1.45)	120 (1.81)	43 (0.65)	48 (0.72)	189 (2.85)	20 (0.3)
February 15, 2020 (n=5383)	81 (1.5)	73 (1.31)	38 (0.71)	35 (0.65)	153 (2.84)	17 (0.32)
March 1, 2020 (n=12,863)	1434 (11.15)	606 (4.71)	245 (1.9)	157 (1.22)	374 (2.91)	41 (0.32)
March 15, 2020 (n=26,550)	3213 (12.1)	427 (1.61)	2275 (8.57)	1226 (4.62)	1966 (7.4)	1091 (4.11)
April 1, 2020 (n=20,772)	1105 (5.32)	323 (1.55)	706 (3.4)	219 (1.05)	789 (3.8)	131 (0.63)
April 15, 2020 (n=21,027)	1022 (4.86)	389 (1.85)	871 (4.14)	198 (0.94)	787 (3.74)	97 (0.46)
May 1, 2020 (n=20,571)	2151 (10.46)	593 (2.88)	795 (3.86)	202 (0.98)	1145 (5.57)	106 (0.52)
May 15, 2020 (n=18,128)	505 (2.79)	293 (1.62)	286 (1.58)	132 (0.73)	654 (3.61)	82 (0.45)

### RQ 5: What Are the Trends in User Participation Across Different Demographics for Each Substance Type?

The monthly trend diagram shown in Figure 11 provides the nuance of user trend in alcohol use by 6 dimensions, namely, overall (or by default), user type, age group, gender, race, and sentiment. The trend for other substance types can be found in Figures S2-S10 in Multimedia Appendix 1. Similarly, the trends of SU (by posts) across 6 dimensions can be found in Figures S11-S20 in Multimedia Appendix 1.

**Figure 11:**
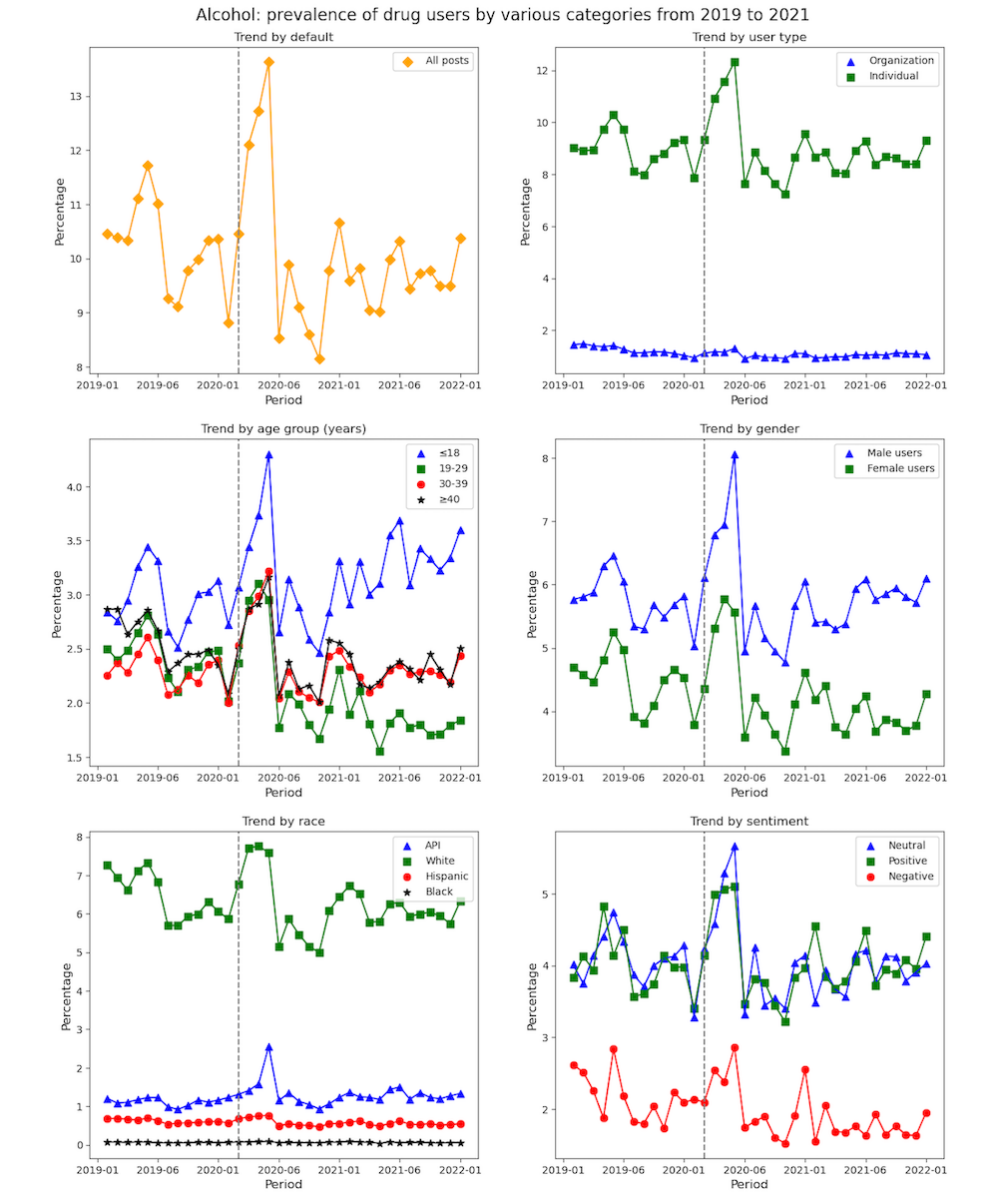
Alcohol users’ distribution across 6 categories from 2019 to 2021. API: Asian or Pacific islander.

### RQ 6: What Emotional Expressions Are Prevalent Across All Substance Types?

**Figure 12:**
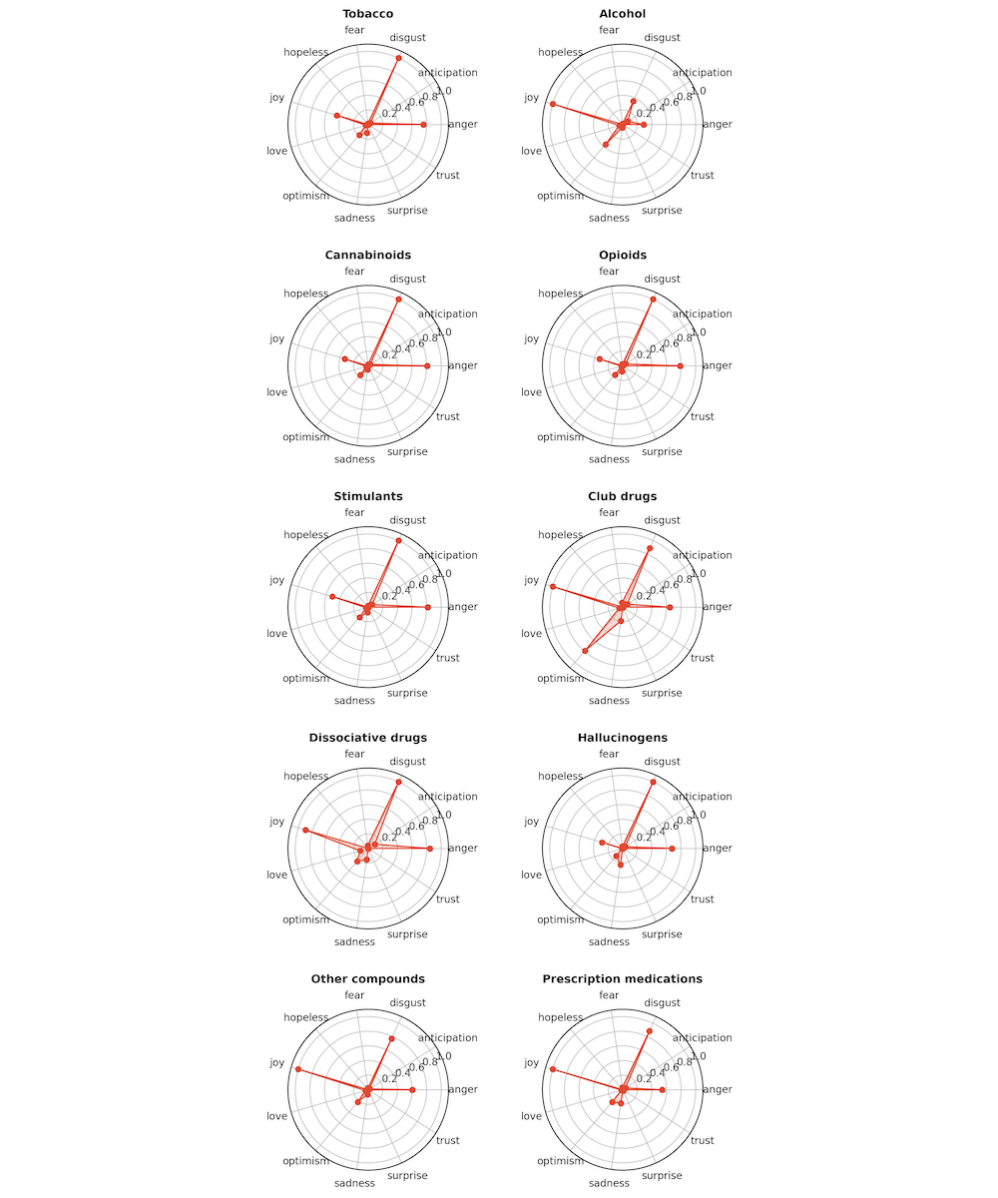
Plutchik emotion analysis across all substance types.

We applied the SpanEmo [35] model to perform emotion detection based on Plutchik Emotion Theory [36], which includes 10 main emotion categories (ie, “anger,” “anticipation,” “disgust,” “fear,” “hopeless,” “joy,” “love,” “optimism,” “sadness,” “surprise,” and “trust”). The detected emotions were processed further to calculate the mean intensity scores, which are presented in the radar plot in Figure 12. In our results, we present emotions for each substance type. Our results showed that SU-related discussions most frequently expressed emotions of joy, disgust, and anger.

## Discussion

### Principal Findings

This study established a foundation for analyzing SU across different demographics using web data, with a particular focus on the COVID-19 pandemic year. In addition to the substantial findings as a result, we made a comprehensive comparison with existing survey-based reports from NCDAS and other research works. Successively, our work has found a notable alignment with survey-based reports, as discussed in RQ 2. Notably, users’ involvement in SU-related discussions surged in 2020, with users increasing by 22.18% compared to 2019 and 25.24% compared to 2021. Demographically, male users overtook female users in discussions, with their share of posts increasing from 48.87% before the pandemic to 53.4% after the pandemic. The youngest age group (aged ≤18 years) remained the most active, with their proportion growing over time, from 39.56% in 2019 to 41.72% in 2021. Among posts with identified racial and ethnic data (64.7%-66.5% of total posts), White users predominated; however, this likely reflects platform demographics and limitations in inference methods rather than actual population distributions.

Each year, cannabinoids, alcohol, stimulants, and tobacco were the most frequently discussed substances (in ascending order), while dissociative drugs and hallucinogens were the least discussed. An overall annual decline was observed across all top substances, except for opioids, which showed a 20% drop only in 2020. A demographic breakdown for 2020 revealed that adults (aged >18 years) and male users dominated discussions on most substances. However, prescription medications and other compounds (edible substances) were more commonly discussed by female users, while tobacco use was more prevalent among teenagers (aged ≤18 years).

An increase in alcohol users was observed following the global pandemic declaration. In just a 2-week period, the alcohol users grew by 80%. Most male teenagers (aged ≤18 years) were involved in alcohol discourse, which is also supported by 2 studies [45,48]. Both studies highlighted that alcohol consumption increased during the peak pandemic lockdown period, driven by factors such as mental health challenges, social isolation, COVID-19–related stress, boredom, and easy availability. These findings are supported by our thematic analysis of alcohol-related discussions.

Another remarkable pattern was observed in the discussion of prescription medication, where female users were more involved in social media discourse. This finding is supported by Peteet et al [28], who reported that female users are more likely to use prescription medication compared to other recreational drugs.

Furthermore, our emotion analysis revealed that alcohol was the only substance strongly associated with the emotion of joy, while all other substances were mostly linked to emotions of disgust and anger. This suggests that alcohol use may be driven by positive or celebratory motives, while other substances are more often associated with negative emotional contexts.

### Interpretation and Public Health Implications

#### Overview

This study provides critical insights into SU discourse on Twitter during the COVID-19 pandemic, revealing key demographic trends, emotional associations, and substance-specific patterns. By contextualizing these findings within sociocultural, behavioral, and pandemic-specific factors, we offer actionable strategies for public health stakeholders to design targeted interventions and policies.

#### Teenagers (Aged ≤18 Years): Early Exposure and Prevention

The youngest age group demonstrated the highest engagement in discussions about alcohol, tobacco, and cannabinoids. This trend may be attributed to increased screen time during lockdowns, amplified peer influence via social media, and the normalization of SU in popular culture. These findings underscore the need for age-specific prevention programs, such as school-based interventions, peer education initiatives, and social media campaigns tailored to teenagers. Addressing the accessibility of substances and providing structured recreational activities during crises are essential to reduce early exposure and experimentation.

#### Male Users: Gender Norms and Coping Mechanisms

Male users dominated discussions about alcohol and opioids, potentially reflecting sociocultural norms that associate SU with masculinity, as well as behavioral responses to pandemic-related stress. Men may have turned to substances as coping mechanisms due to societal expectations that discourage emotional expression and help-seeking behaviors. Male-centric messaging should emphasize healthy coping strategies and challenge harmful gender norms. Community-based programs, such as sports and recreational activities, can provide alternative outlets for stress relief and reduce reliance on substances. This aligns with NSDUH gender disparities but may be amplified by Twitter’s male-skewed user base (estimated 62% male users in 2021).

#### Female Users: Prescription Medication Misuse

Female users were more likely to engage in discussions about prescription medications, aligning with existing research on higher rates of prescription drug misuse among women. This pattern may be influenced by higher rates of chronic pain conditions, greater likelihood of being prescribed medications, and pandemic-related stressors such as increased caregiving responsibilities. Gender-specific educational programs should address the risks of prescription drug misuse and promote alternative pain management strategies. Health care providers should be trained to recognize and address gender-specific risk factors during patient consultations.

#### Alcohol: Positive Perceptions and Social Coping

Alcohol was uniquely associated with the emotion of joy, suggesting it is often perceived positively and used as a social and coping mechanism. This perception may be reinforced by social media content that glamorizes alcohol consumption. Public health campaigns should reframe societal perceptions of alcohol, highlighting its negative health consequences and promoting nonalcoholic alternatives for stress relief and social interaction. Policies limiting alcohol accessibility, particularly for underage individuals, should also be considered.

#### Opioids and Other Substances: Distress and Self-Medication

Substances such as opioids, cannabinoids, and stimulants were linked with negative emotions, indicating that their use may be driven by distress or self-medication for underlying mental health issues. Harm reduction strategies, such as increasing access to addiction treatment services and mental health support, are critical. Integrating mental health screening into SU prevention programs can help identify individuals considered to be at risk and provide early intervention.

#### Leveraging Social Media for Public Health

This study highlights the potential of social media as a real-time surveillance tool for monitoring SU trends. Platforms such as Twitter can be leveraged to disseminate prevention messages, identify emerging trends, and engage populations considered to be at risk. Policies such as stricter alcohol regulations for underage individuals and enhanced prescription drug monitoring programs can further mitigate substance misuse. Integrating mental health support into SU prevention and treatment programs is also essential, given the strong link between negative emotions and SU.

By connecting these patterns to sociocultural, behavioral, and contextual factors, this study not only advances the understanding of SU discourse during the COVID-19 pandemic but also provides a road map for public health stakeholders to design targeted interventions, policies, and campaigns. Future research should explore the integration of multiplatform data and multilingual analyses to further enhance the generalizability and applicability of these findings.

### Limitations

This study builds upon our previous research [37]. As in our previous research, there is data skewness in certain months due to missing data in the original source [39]. This could potentially deviate from the actual results. Likewise, the SU identifier developed in our previous research [37], which used advanced deep learning tools such as RoBERTa and human-in-the-loop methods, achieved an accuracy of 80%. Thus, the user base analysis studied in this study does not account for all the substance users.

Second, we recognize that our study’s focus on English-language posts may have limited the generalizability of our findings. By excluding non–English-speaking populations, we may have overlooked diverse racial groups and specific age groups in SU discourse. The focus on English-language content, combined with the lack of geocoding analysis, likely overrepresents English-speaking regions, particularly the United States, while underrepresenting global SU patterns. In addition, the user base identified in the study does not investigate the frequency of posts. Analyzing retweeted posts or frequent users could reveal deeper insights that may help public health policy makers develop more targeted and effective strategies.

Third, for the demographic identification, we relied on various machine learning models, such as m3inference and EthicolrM. Although we validated the tools with our ground data, the bias in these models still exists. For age and gender, we only achieve 80% validation accuracy using the m3inference model, which perhaps is due to the specific training dataset that did not fully capture the diversity of Twitter users. For race, our Ethicolr-based pipeline classified only on average of 65% (4.3 million/6.6 million) of users across study years, with unclassified users potentially skewing results if racial groups differed in their likelihood of providing identifiable metadata. This limitation is compounded by the model’s training on US census data, which may not generalize to global or non-Anglicized naming conventions, and our reliance on only English-language posts. Thus, we acknowledge that these tools may have introduced biases in the extraction of age, gender, and race. This could have potentially affected the representativeness and accuracy of our findings.

Fourth, we acknowledge that the observed demographic trends (eg, male vs female participation) may be confounded by the inherent demographic distribution of Twitter users. Normalizing these trends by Twitter user base proportions for each demographic could provide a more accurate representation; however, such data are not publicly available, limiting our ability to perform this analysis.

Fifth, our analysis is limited to a single platform, Twitter, which might not fully represent the broader spectrum of SU discourse across other social media platforms such as Facebook, Instagram, TikTok, and Reddit.

Sixth, we acknowledge limitations regarding comparative analyses with survey data. While our findings show meaningful alignments with reports such as NSDUH and NCDAS, important methodological differences must be considered: our real-time social media data capture immediate discourse rather than the annualized behaviors reported in surveys; clinical surveys use validated screening tools such as Alcohol Use Disorders Identification Test, whereas our classifier detects broader public discourse, including news and advocacy content; and NSDUH’s nationally representative sampling contrasts with Twitter’s self-selected user base. These factors suggest that our temporal trends should be interpreted as complementary to, rather than confirmatory of, survey findings.

Finally, we acknowledge the limitation of the studied themes. While we referenced key COVID-19 factors derived from our primary study [37], the trends could have been influenced by other societal factors such as political tensions. Furthermore, the keywords used in identifying themes may have been too narrow, potentially leading to an overrepresentation of certain themes in our results.

### Future Work

This study focused on analyzing SU differences from the perspectives of age, gender, and race. To enhance understanding, future work could incorporate geolocation data to analyze trends and patterns, enabling exploration of region-specific influencing factors. This would enable the development of targeted intervention strategies to prevent SU based on geographic location. Furthermore, extracting additional information, such as socioeconomic, mental, and physical health status, could significantly enhance the use of social media as a prominent platform for studying public health–related issues. In addition, analyzing user-based personality traits could provide valuable insights for the public health sector, allowing for the identification of specific characteristics that can inform prevention strategies, even in the absence of demographic information.

To address the limitations identified in this study, future research could also expand the analysis to include multiple languages, leveraging multilingual NLP models to capture a more comprehensive understanding of SU discourse across diverse linguistic and cultural contexts. Developing and using more robust, inclusive, and transparent demographic inference models trained on diverse datasets would further enhance the representativeness of findings. Future studies could also explore normalizing demographic trends by the underlying user base proportions of social media platforms, if such data become available. This would provide a more accurate representation of demographic participation and strengthen the generalizability of findings. In addition, expanding the scope to include other social media platforms (eg, Facebook, Reddit, and Instagram) and offline data sources (eg, surveys and interviews) would provide a more holistic view of SU discourse during public health crises. Collaborations with researchers fluent in non-English languages, as well as social scientists and ethicists, could help refine these tools and methodologies to better account for intersectional identities, cultural contexts, and global perspectives.

### Conclusions

Social media platforms, combined with advanced NLP technologies, offer a valuable alternative research space for uncovering insightful trends and patterns in SU discourse. This study has successfully demonstrated the potential of leveraging Twitter’s data to analyze SU trends during the COVID-19 pandemic, aligning our findings with notable survey-based reports, such as NCDAS and Monitoring the Future. Our results highlight significant demographic shifts, such as the increased engagement of teenagers and male users in substance-related discussions, as well as substance-specific patterns, including the rise in alcohol discourse and the gender disparities in prescription medication discussions.

These insights offer actionable strategies for public health stakeholders, enabling targeted interventions for groups considered to be high risk and substance-specific harm reduction. By leveraging social media as a real-time surveillance tool, stakeholders can monitor trends, disseminate prevention messages, and engage populations considered to be at risk without relying on traditional surveying methods. This study underscores the potential of social media data to inform public health strategies, particularly during global crises, and emphasizes the need for future research to include multiple platforms and languages to enable more inclusive and impactful interventions.

## Appendix

Multimedia Appendix 1 Supplementary tables and figures.

## Abbreviations

NCDAS: National Center for Drug Abuse Statistics
NIDA: National Institute on Drug Abuse
NLP: natural language processing
NSDUH: National Survey on Drug Use and Health
RoBERTa: Robustly Optimized Bidirectional Encoder Representations From Transformers Pretraining Approach
RQ: research question
SAMHSA: Substance Abuse and Mental Health Services Administration
SU: substance use
VADER: Valence Aware Dictionary and Sentiment Reasoner

## Data Availability

The data and code supporting this study are publicly available on GitHub [49].
