# Supplementary material for: Differential Analysis of Age, Gender, Race, Sentiment, and Emotion in Substance Use Discourse on Twitter During the COVID-19 Pandemic: A Natural Language Processing Approach
Source: JMIR Infodemiology. 2025 Jul 28;5:e67333. doi: 10.2196/67333 (PMC12340460; doi:10.2196/67333)

## **Multimedia Appendix 1**

Table S1. Gender, age group, and organization prediction result for sample posts (with name, description, screen name) from M3Inference

| **Name** | **Screen name** | **Description** | **Age Group** | **Gender** | **Org** |
| --- | --- | --- | --- | --- | --- |
| Elon Musk | elonmusk | Nan | >=40 | Male | non-org |
| Barak Obama | BarackObama | Dad, husband, President, citizen. | >=40 | Male | non-org |
| Mckenna Grace | MckennaGracefull | Instagram:@mckennagraceful | <=18 | Female | non-org |
| Millie Bobby Brown | Milliestopshate | I want this account to share love and positivity | <=18 | Female | non-org |
| NASA | NASA | There's space for everybody | NAN | NAN | org |

Table S2. Race prediction result for sample posts (First name and Last name) from Ethicolr

| **Name** | **Race** |
| --- | --- |
| Michael Jackson | Black |
| John Smith | White |
| Austin Alderson | White |
| Zhang | API |
| Shakya | API |
| Kendrick Valdez | Hispanic |

Table S3. Sentiment prediction result for sample posts from VADER

| **Tweet** | **Sentiment** |
| --- | --- |
| USER stag drunkenness buzz tipsy drunk hammer nancy pelosi | Negative |
| USER night jump drunk men try brake fight break cartilage nose bone | Negative |
| USER yeah smoker notice smell | Positive |
| USER yeah smoker notice smell | Positive |
| USER thinking nicotine | Neutral |
| USER girl texas tech lose life week ago someone thought could drive drunk mom amp dad lose daughter | Neutral |

Table S4. Top 5 words associated with each emotion class

| **Negative emotions** | |
| --- | --- |
| Anger | death, think, public, virus, don’t, against |
| Disgust | deaths, virus, against, because, public, after |
| Fear | deaths, spread, symptoms, coronavirus, identify, self-reporting |
| Sadness | deaths, going, cases, hospital, other, please |
| Pessimism | sadly, family, friend, during, weeks, passed |
| **Positive emotions** | |
| Anticipation | support, vaccine, first, working, public, cases |
| Joy | great, thank, support, happy, amazing, staysafe |
| Trust | trust, thank, protect, important, community, everyone |
| Love | happy, loved, share, beautiful, wonderful, amazing |
| Optimism | please, thank, support, working, great, spread |
| Surprise | shocking, surprised, amazing, public, absolutely, deaths |

Table S5. Sample prediction result for posts from SpanEmo

| **Tweet** | **Emotions** |
| --- | --- |
| @Adnan__786__ @AsYouNotWish Dont worry Indian army is on its ways to dispatch all Terrorists to Hell | ['anger', 'disgust', 'fear'] |
| Academy of Sciences, eschews the normally sober tone of scientific papers and calls the massive loss of wildlife a “biological annihilation | ['anger', 'disgust', 'sadness'] |
| I blew that opportunity -__- #mad | ['anger', 'disgust'] |

Table S6. Distribution of Substance Use Discourse Aggregated by Post and User

|  | **Pre-Pandemic (2019)** | | **During Pandemic (2020)** | | **Post-Pandemic (2021)** | |
| --- | --- | --- | --- | --- | --- | --- |
|  | **By Posts** | **By Users** | **By Posts** | **By Users** | **By Posts** | **By Users** |
| **Total SU Counts** | 2,799,726 | 2,131,457 | 3,502,171 | 2,604,123 | 2,553,235 | 1,946,742 |
| **User type** |  | | | | | |
| Org | 221,934 (7.93%) | 153,779 (7.21%) | 291,136 (8.31%) | 195,648 (7.51%) | 232,207 (9.09%) | 159,816 (8.21%) |
| Person | 2,577,792 (92.07%) | 1,977,678 (92.79%) | 3,211,035 (91.69%) | 2,408,475 (92.49%) | 2,321,028 (90.91%) | 1,786,926 (91.79%) |
| **Gender Type** |  | | | | | |
| Female | 1,318,063 (51.13%) | 1,007,368 (50.94%) | 1,558,219 (48.53%) | 1,166,535 (48.43%) | 1,081,682 (46.60%) | 828,102 (46.34%) |
| Male | 1,259,729 (48.87%) | 970,310 (49.06%) | 1,652,816 (51.47%) | 1,241,940 (51.57%) | 1,239,346 (53.40%) | 958,824 (53.66%) |
| **Age Group** |  | | | | | |
| <=18 | 1,019,817 (39.56%) | 793,067 (40.10%) | 1,257,269 (39.15%) | 970,641 (40.30%) | 968,414 (41.72%) | 759,859 (42.52%) |
| 19-29 | 772,388 (29.96%) | 587,274 (29.70%) | 849,019 (26.44%) | 633,760 (26.31%) | 542,770 (23.38%) | 419,018 (23.45%) |
| 30-39 | 468,354 (18.17%) | 361,423 (18.28%) | 638,334 (19.88%) | 476,802 (19.80%) | 488,024 (21.03%) | 370,951 (20.76%) |
| >=40 | 317,233 (12.31%) | 235,914 (11.93%) | 466,413 (14.53%) | 327,272 (13.59%) | 321,820 (13.87%) | 237,098 (13.27%) |
| **Sentiment** |  | | | | | |
| Neutral | 985,973 (35.22%) | 752,499 (35.30%) | 1,254,059 (35.81%) | 933,994 (35.87%) | 938,201 (36.75%) | 720,580 (37.01%) |
| Positive | 814,611 (29.10%) | 622,641 (29.21%) | 1,006,366 (28.74%) | 748,546 (28.74%) | 788,800 (30.89%) | 592,765 (30.45%) |
| Negative | 999,142 (35.69%) | 756,317 (35.48%) | 1,241,746 (35.46%) | 921,583 (35.39%) | 826,234 (32.36%) | 633,397 (32.54%) |
| **Race*** |  | | | | | |
| Total Race identified | 1,811,516 (64.70%) | 1,811,516 (64.70%) | 2,275,943 (64.98%) | 2,275,943 (64.98%) | 1,723,470 (67.50%) | 1,723,470 (67.50%) |
| API | 303,500 (11.77%) | 272,093 (13.76%) | 406,301 (12.65%) | 363,338 (15.09%) | 302,074 (13.01%) | 277,011 (15.50%) |
| White | 1,215,550 (47.15%) | 1,114,892 (56.37%) | 1,506,359 (46.91%) | 1,367,638 (56.78%) | 1,142,020 (49.20%) | 1,053,655 (58.96%) |
| Hispanic | 126,121 (4.89%) | 117,406 (5.94%) | 148,930 (4.64%) | 136,620 (5.67%) | 107,018 (4.61%) | 99,447  (5.57%) |
| Black | 12,596  (0.49%) | 11,731  (0.59%) | 16,045  (0.50%) | 14,879 (0.62%) | 12,068  (0.52%) | 11,299  (0.63%) |
| Unidentified | 988,210 (35.29%) | 988,210 (35.29%) | 1,226,228 (35.01%) | 1,226,228 (35.01%) | 829,765 (32.49%) | 829,765 (32.49%) |

**Note:**

1. The **Total SU Counts** identified varied across years due to fluctuations in the number of posts and users. Consequently, changes in proportions do not necessarily reflect a consistent change in the weighted count.
2. The notation (*****) in Race* indicates that the proportion within the subgroup does not account for the base total of tweets. This is because not all posts included a valid username that could be used to infer race or ethnicity, leading to a reduction in the total number of posts after race identification.

Figure S1: Alcohol Distribution by demographics in 2019, 2020 and 2021


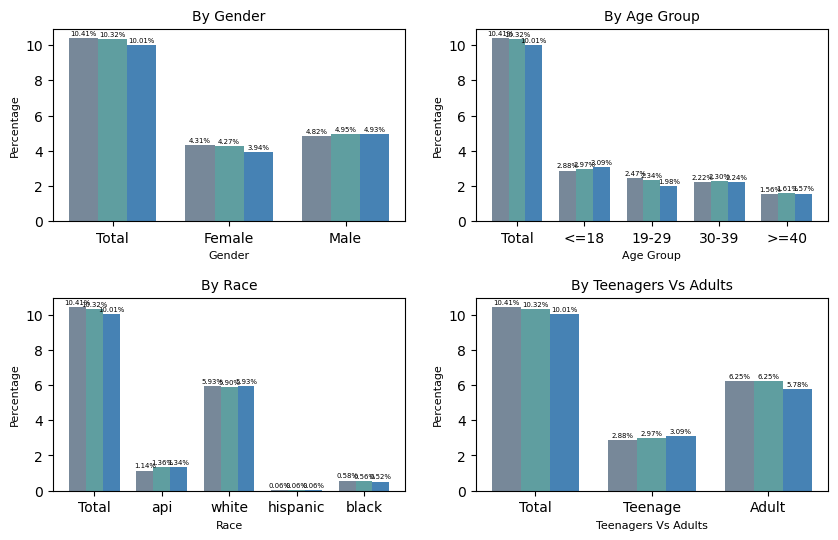


##

Figure S2. Tobacco user distribution across six categories from 2019 to 2021


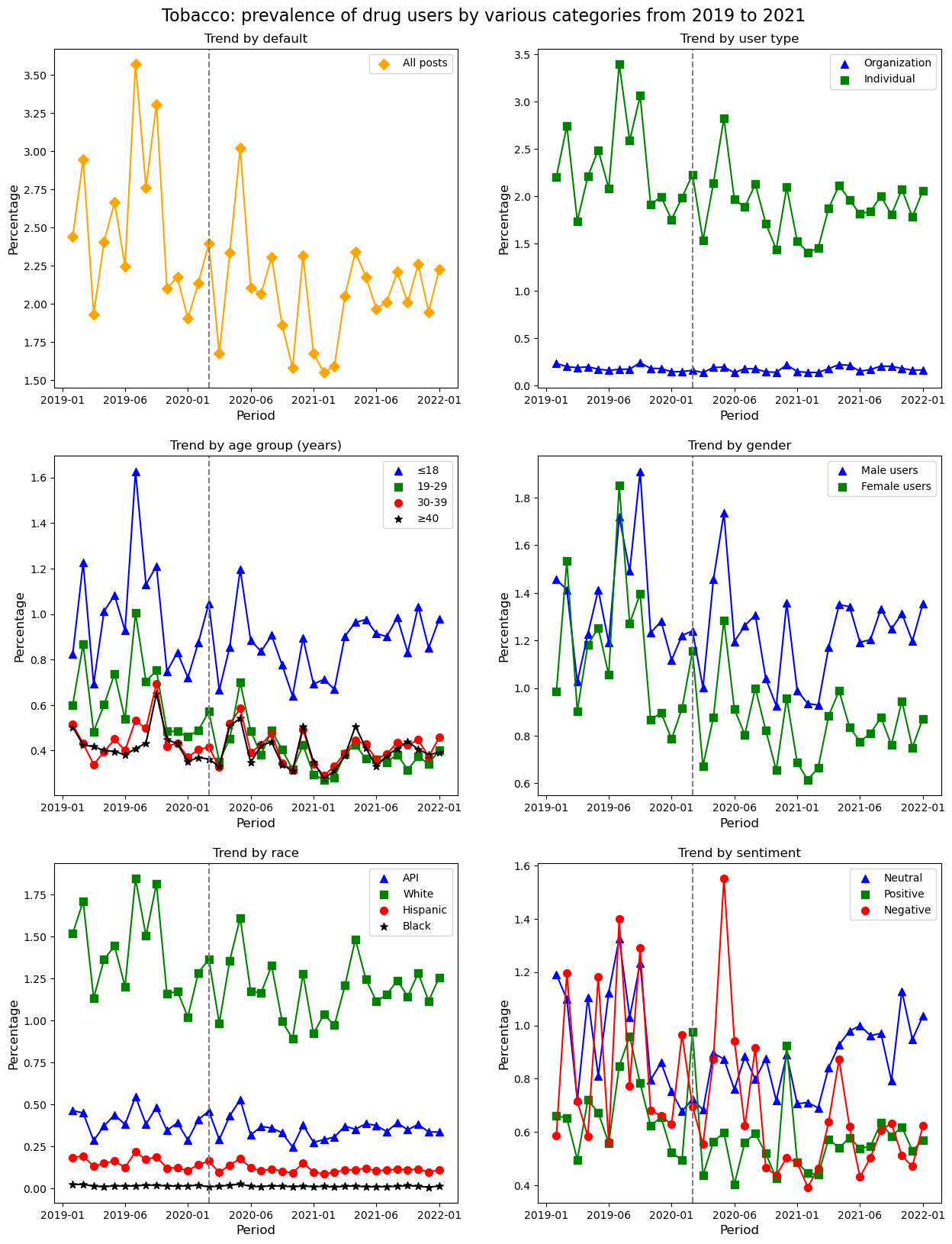


Figure S3. Cannabinoids user distribution across six categories from 2019 to 20
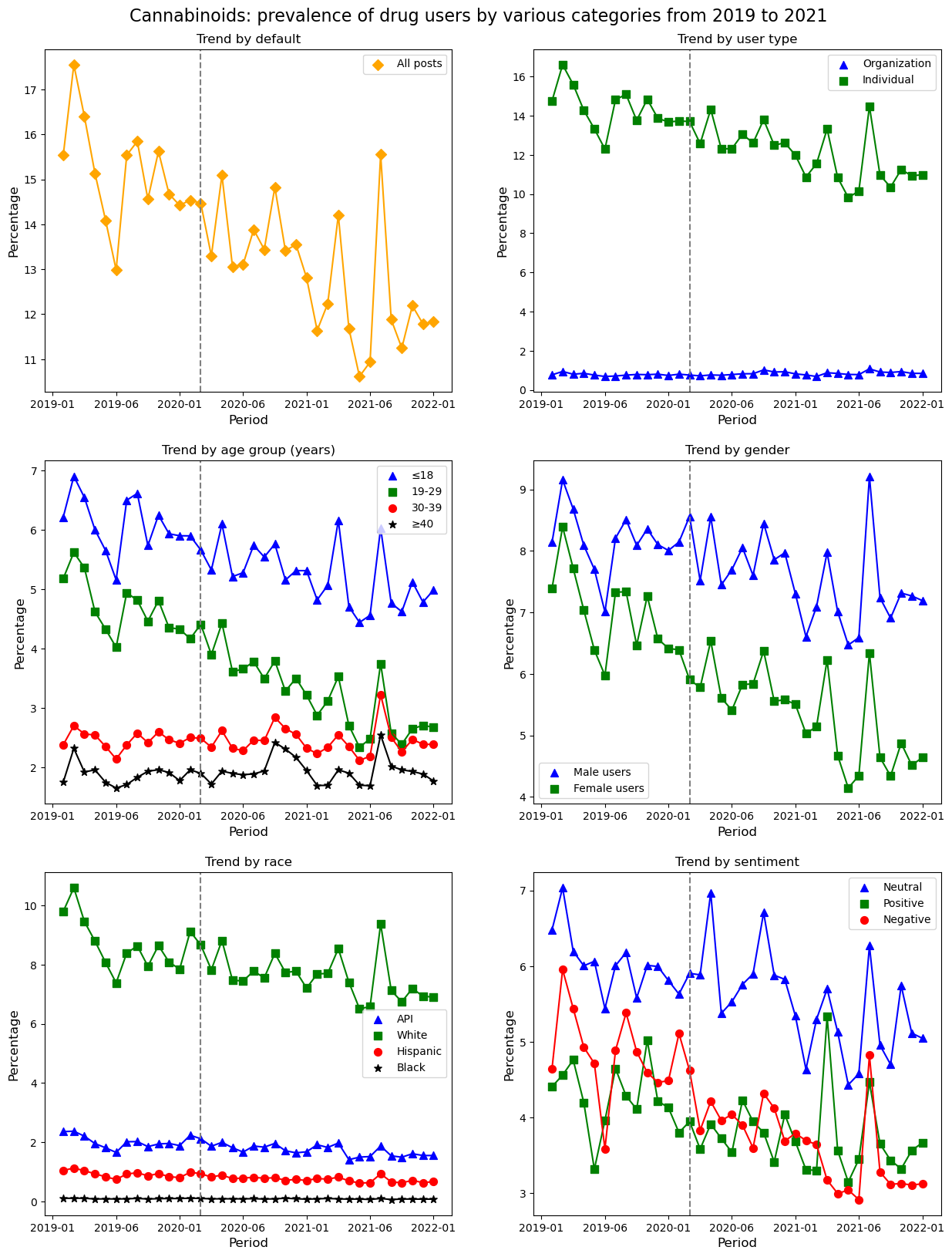


Figure S4. Opioids user distribution across six categories from 2019 to 2021


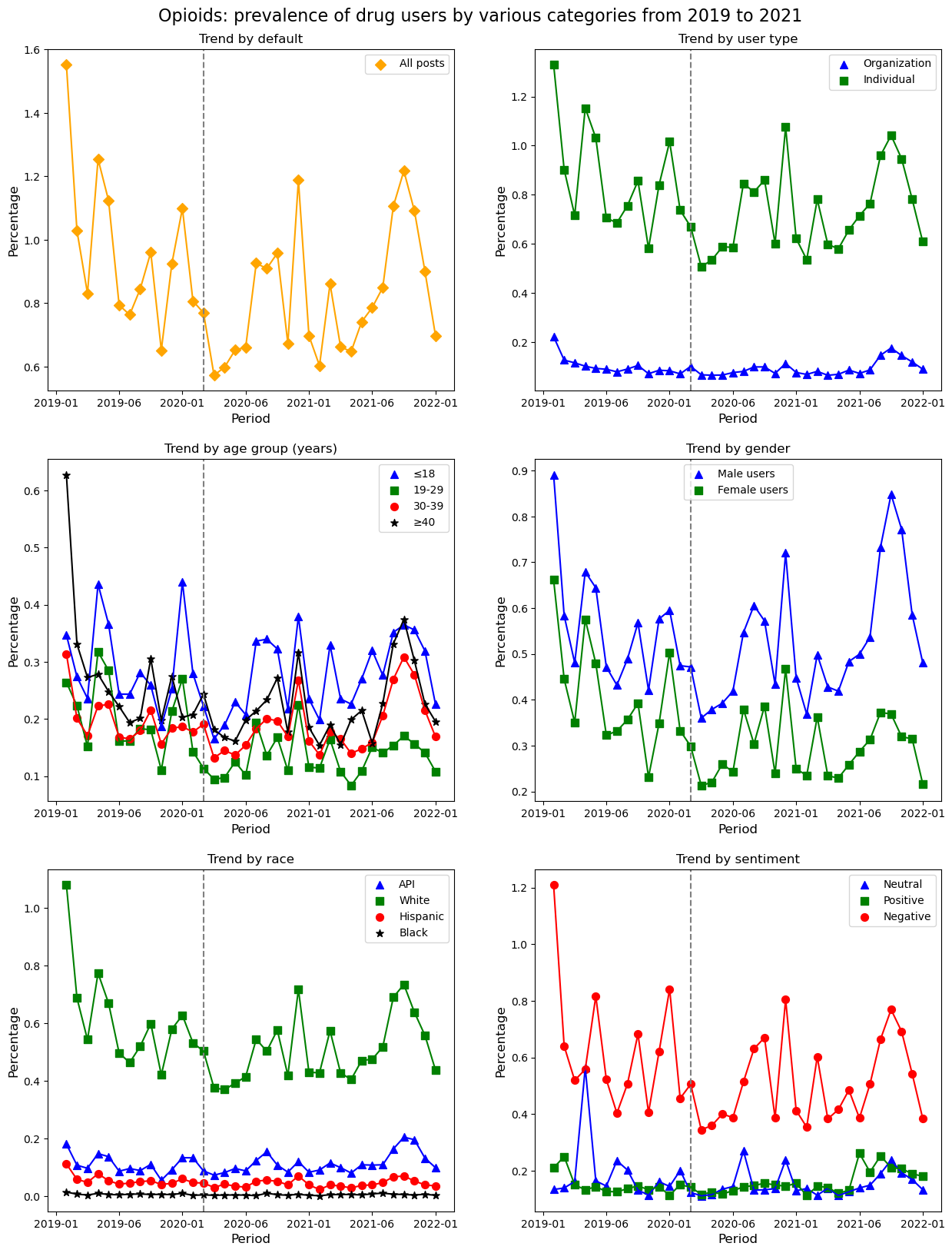


Figure S5. Stimulants user distribution across six categories from 2019 to 2021


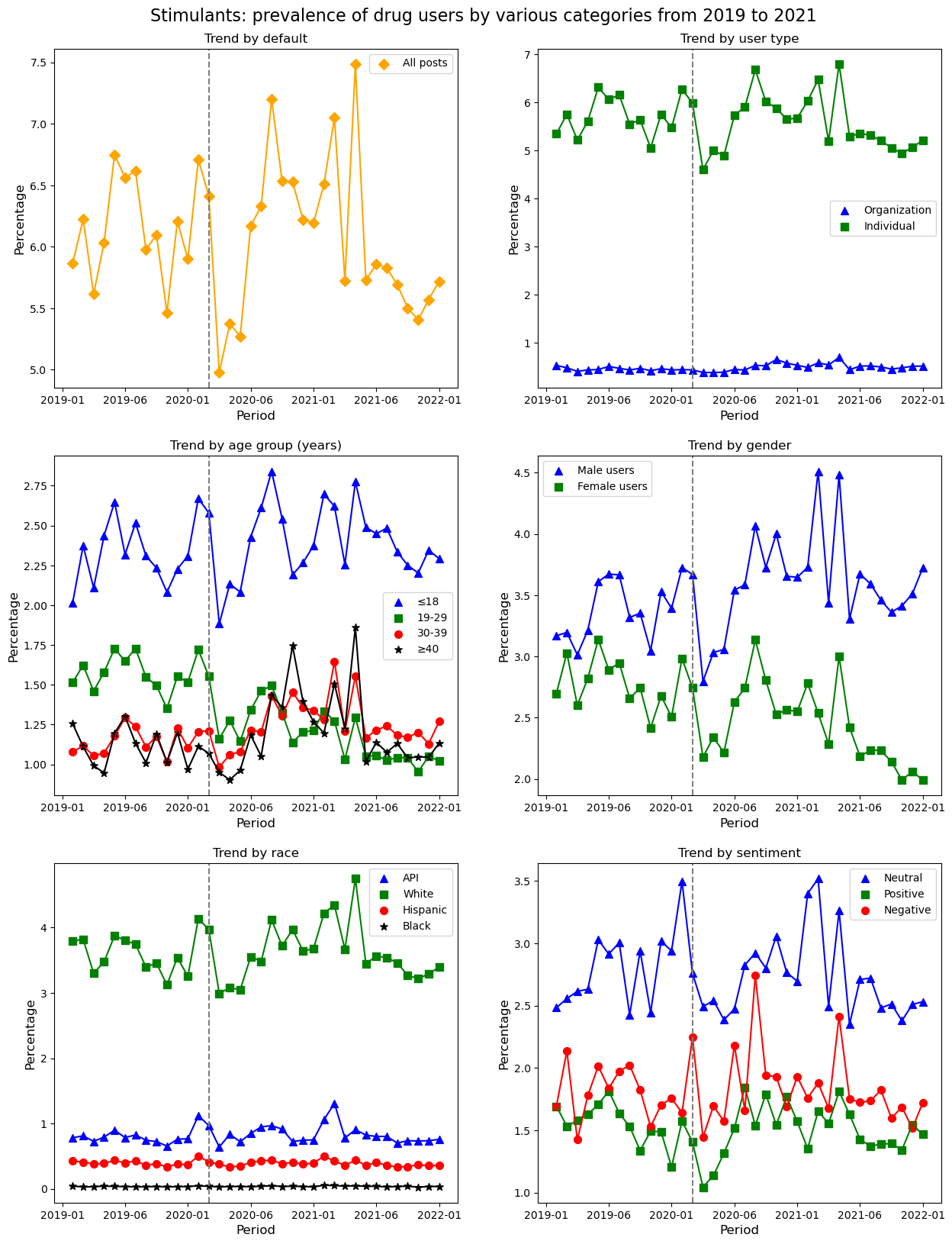


Figure S6. Club Drugs user distribution across six categories from 2019 to 2021
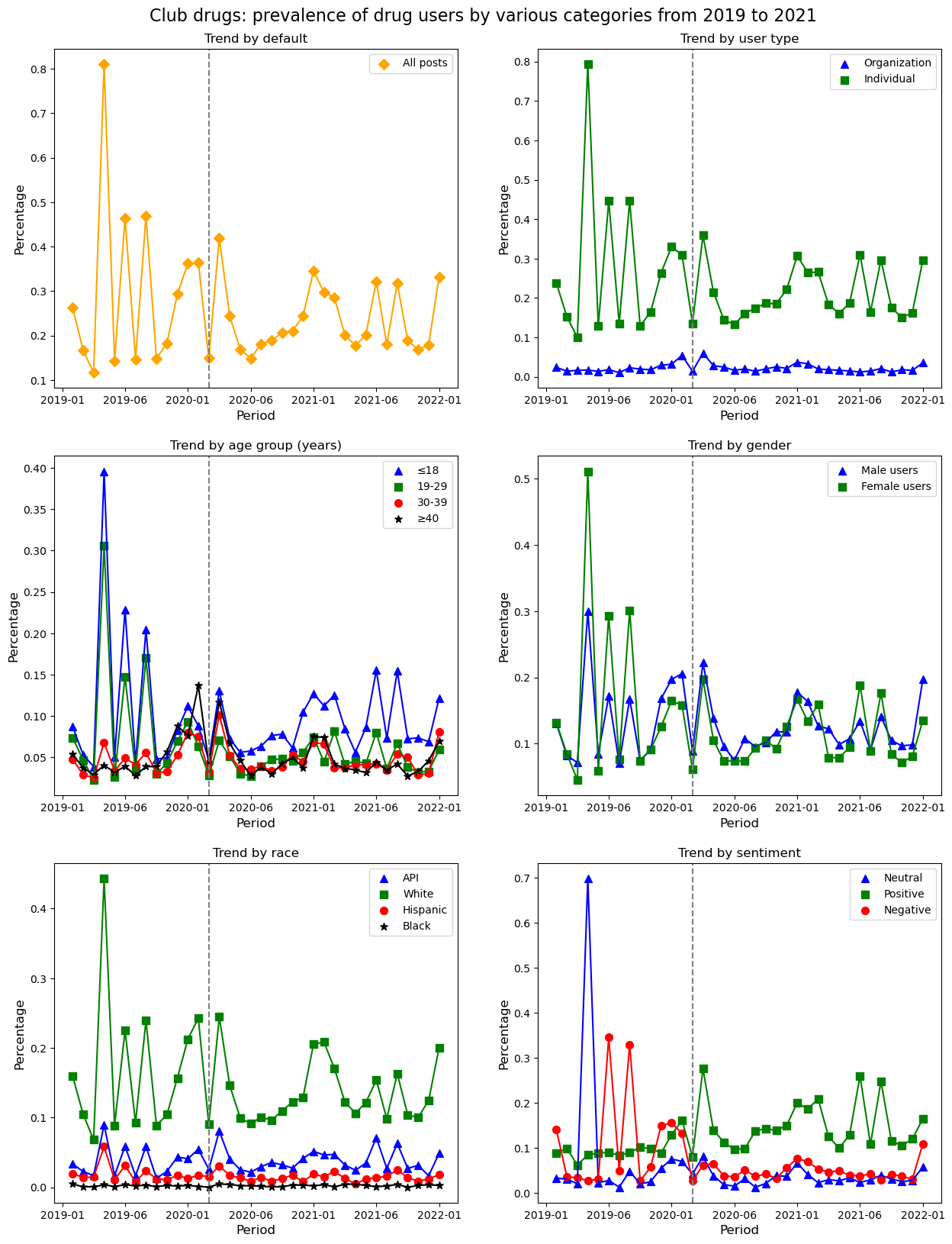


Figure S7. Dissociative user distribution across six categories from 2019 to 2021


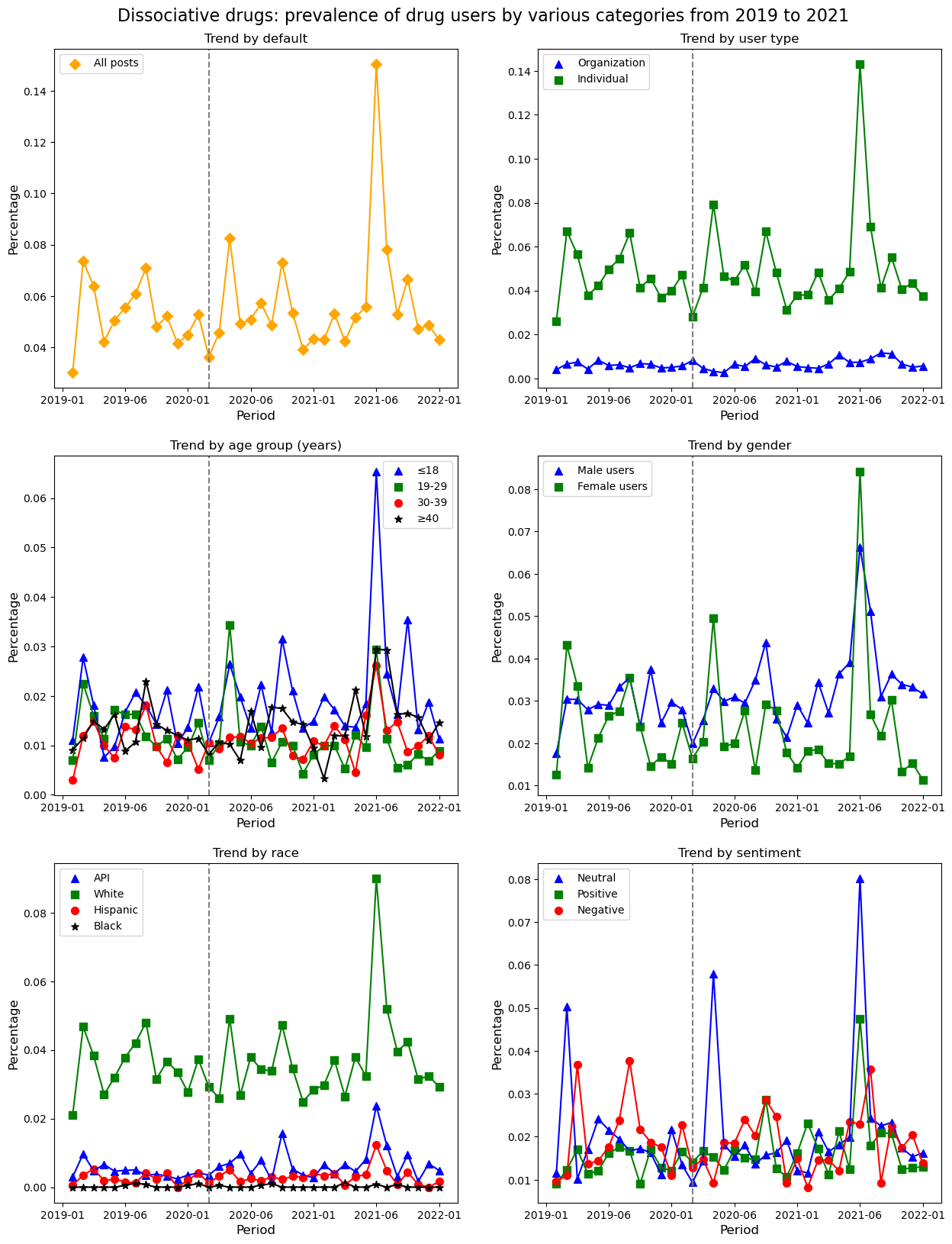


Figure S8. Hallucinogens user distribution across six categories from 2019 to 2021


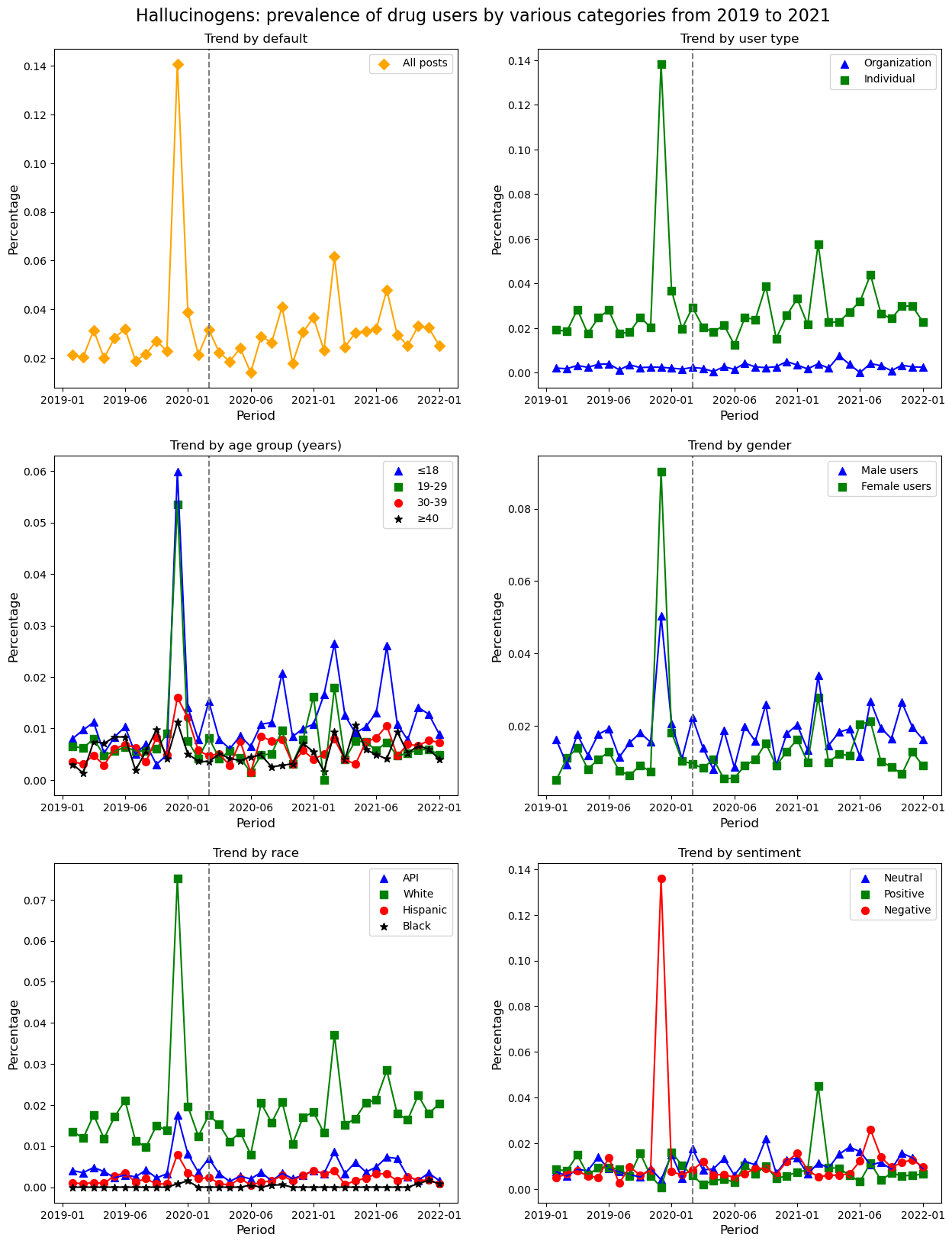


Figure S9. Other Compounds user distribution across six categories from 2019 to 2021
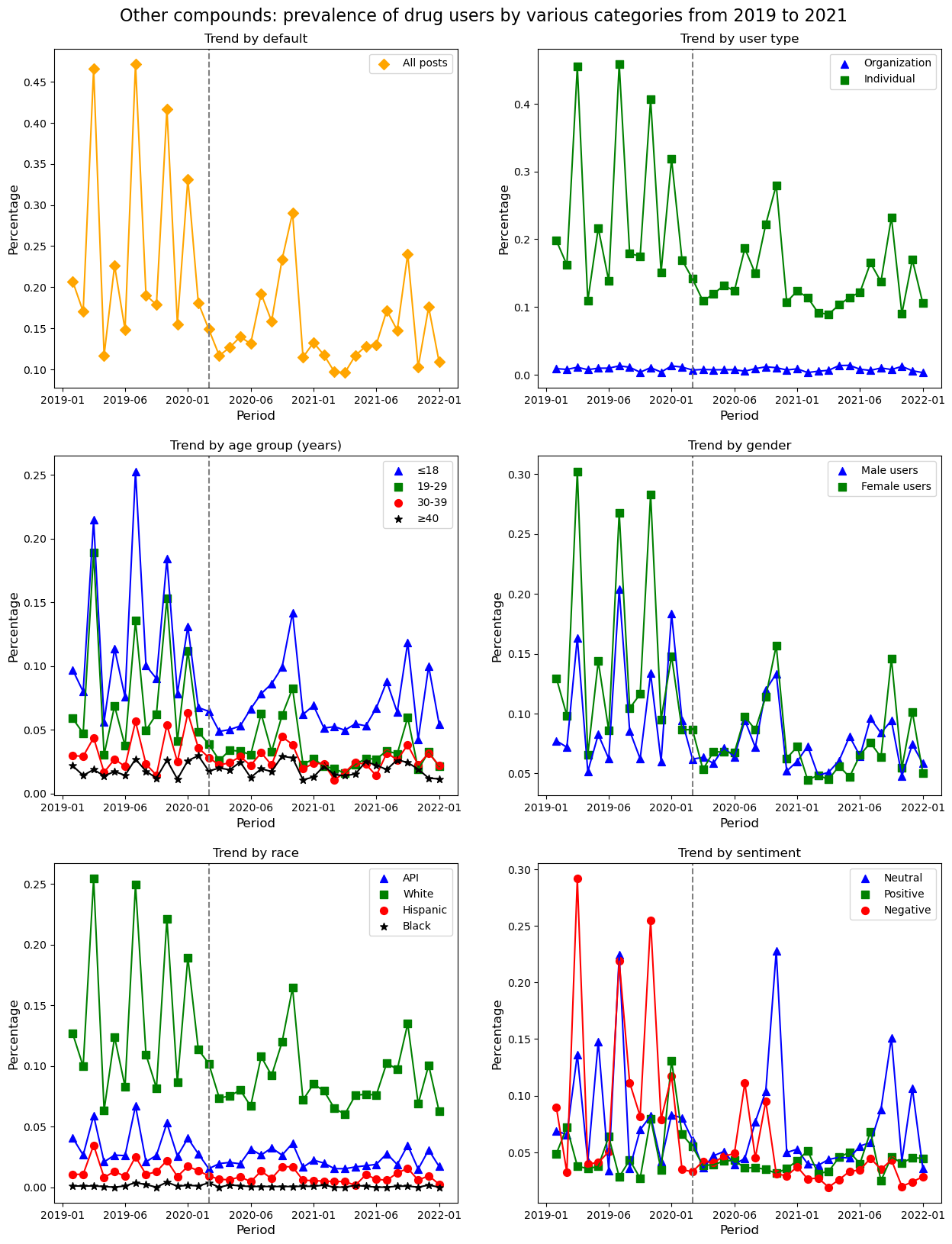


Figure S10. Prescription Medications user distribution across six categories from 2019 through 2021
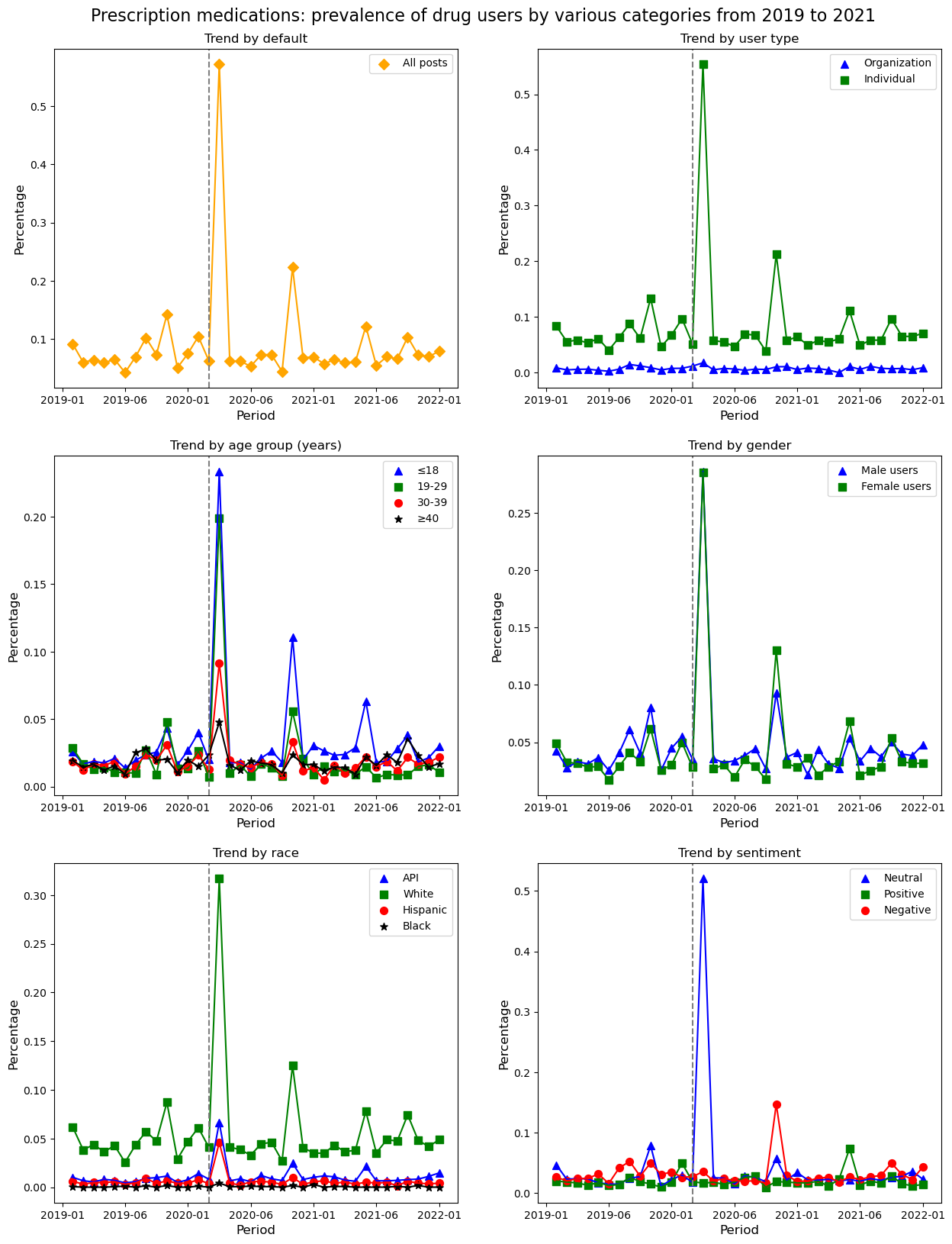


Figure S11. Tobacco posts distribution across six categories from 2019 to 2021


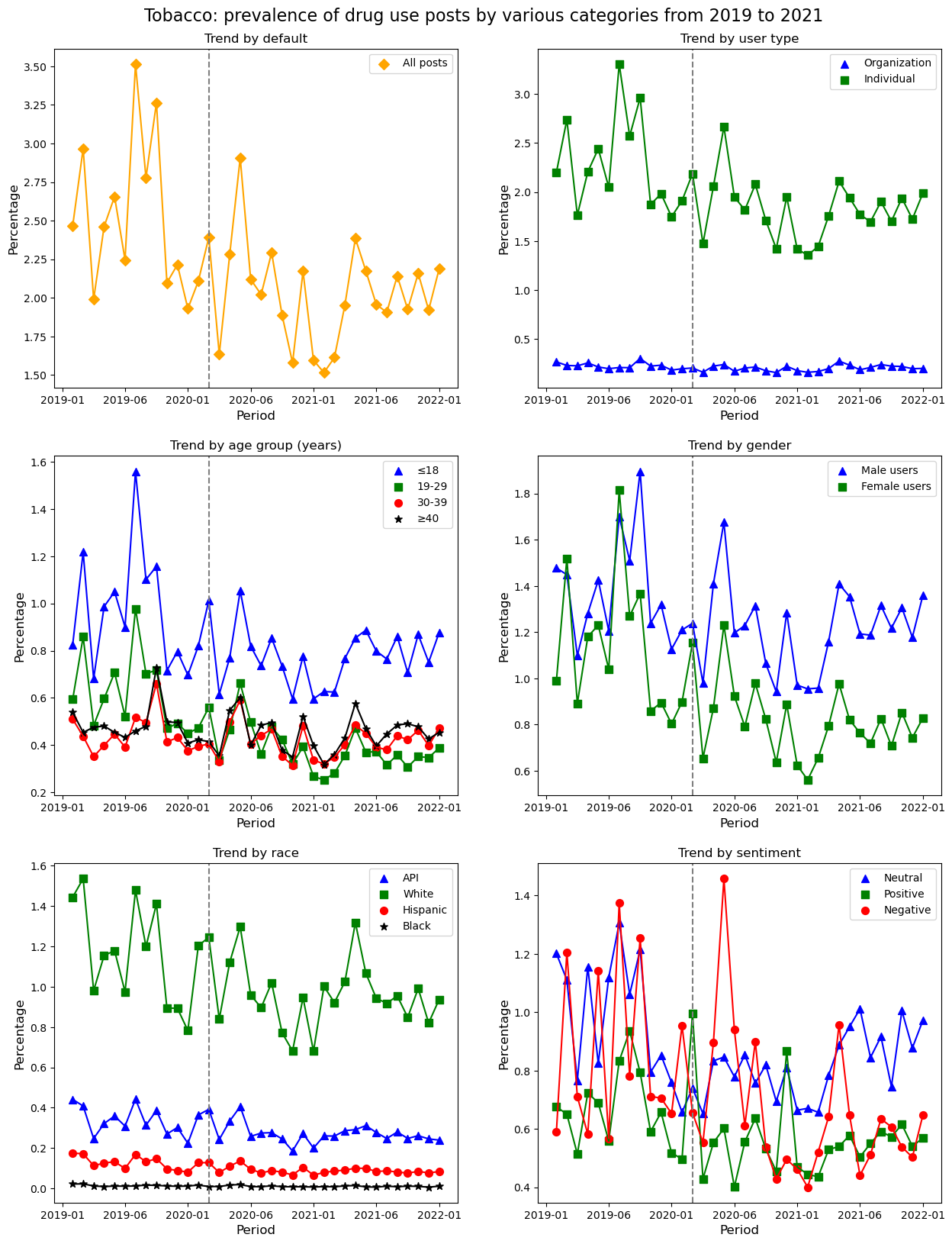


Figure S12. Alcohol posts distribution across six categories from 2019 to 2021


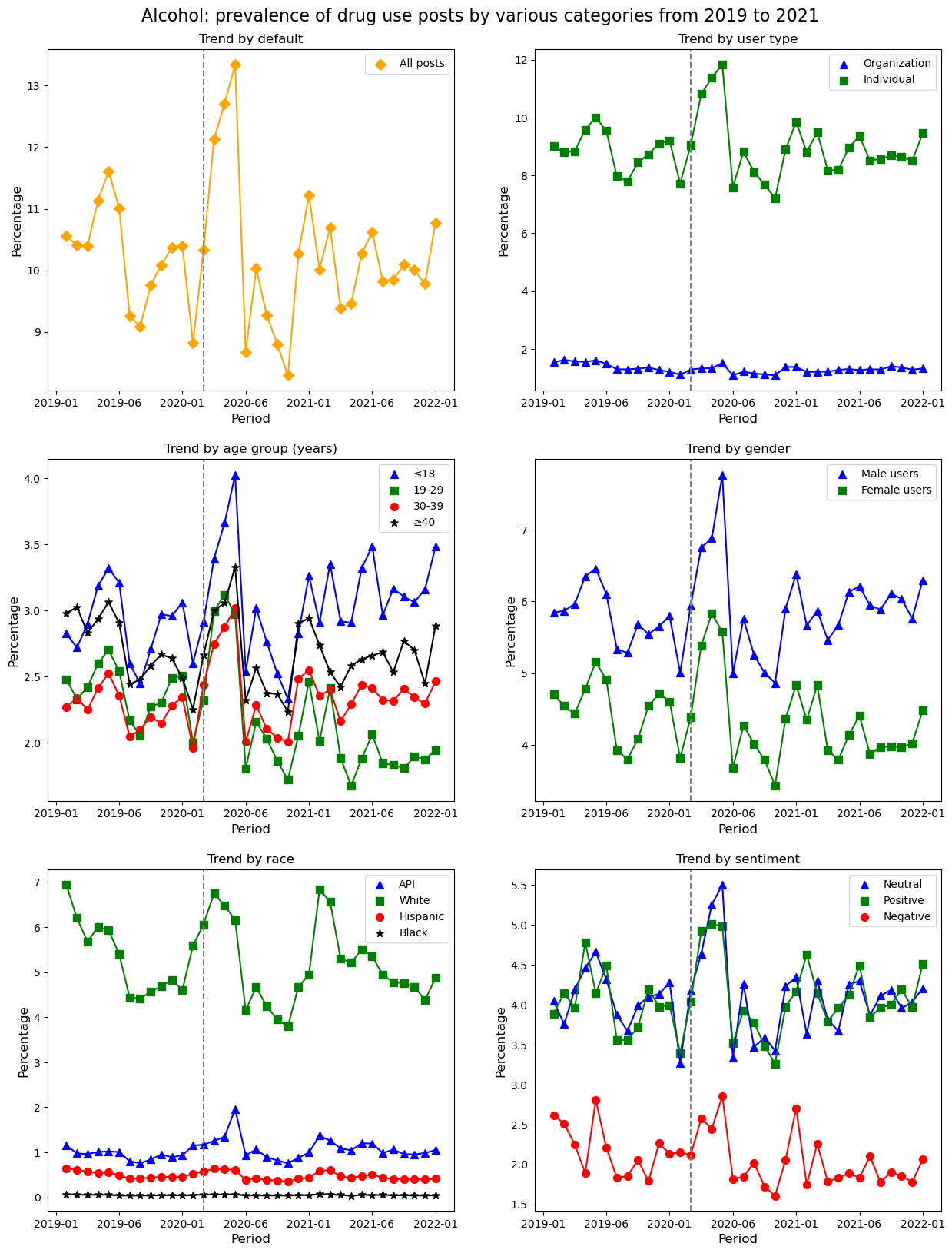


Figure S13. Cannabinoids posts distribution across six categories from 2019 to 2021


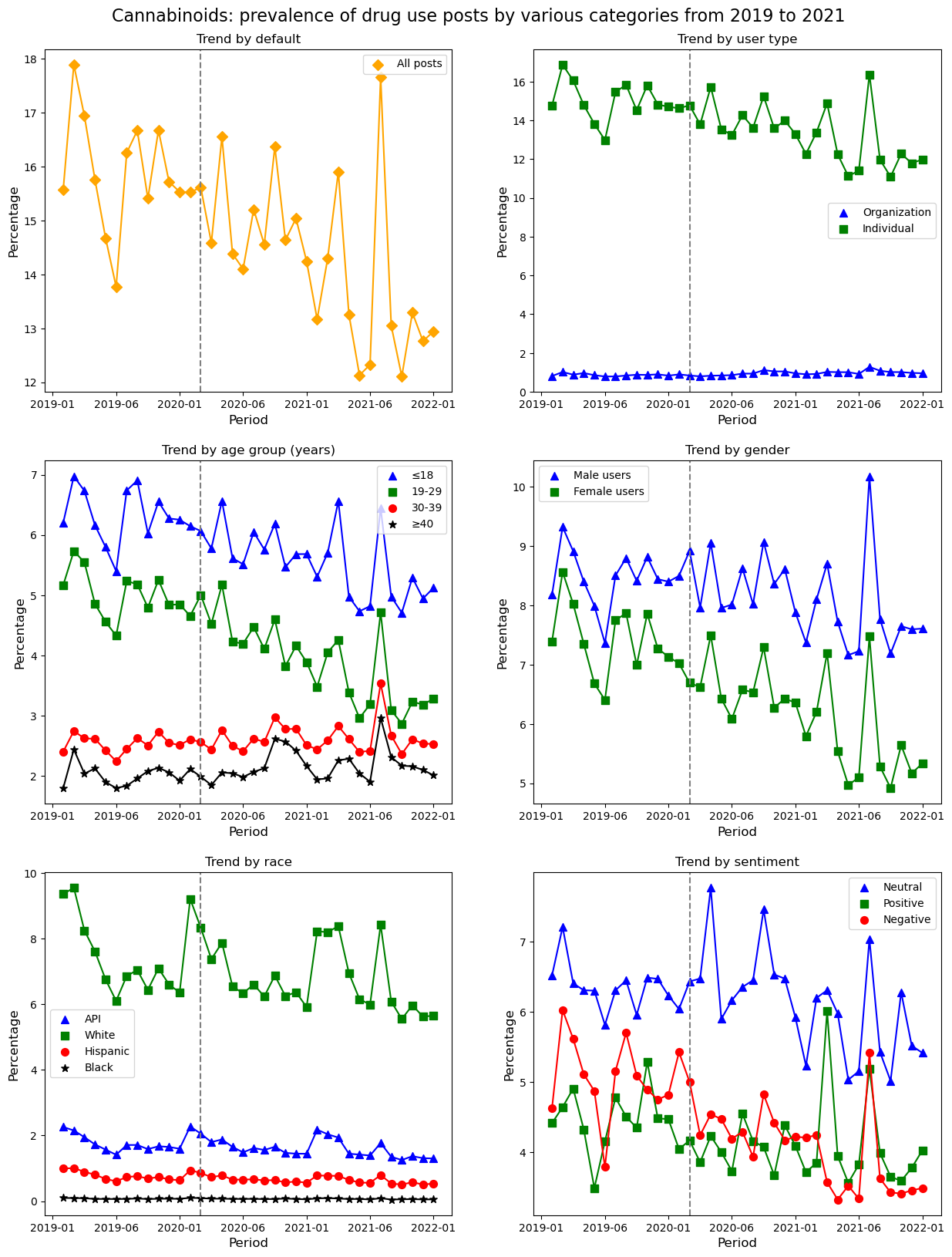


Figure S14. Opioids posts distribution across six categories from 2019 to 2021


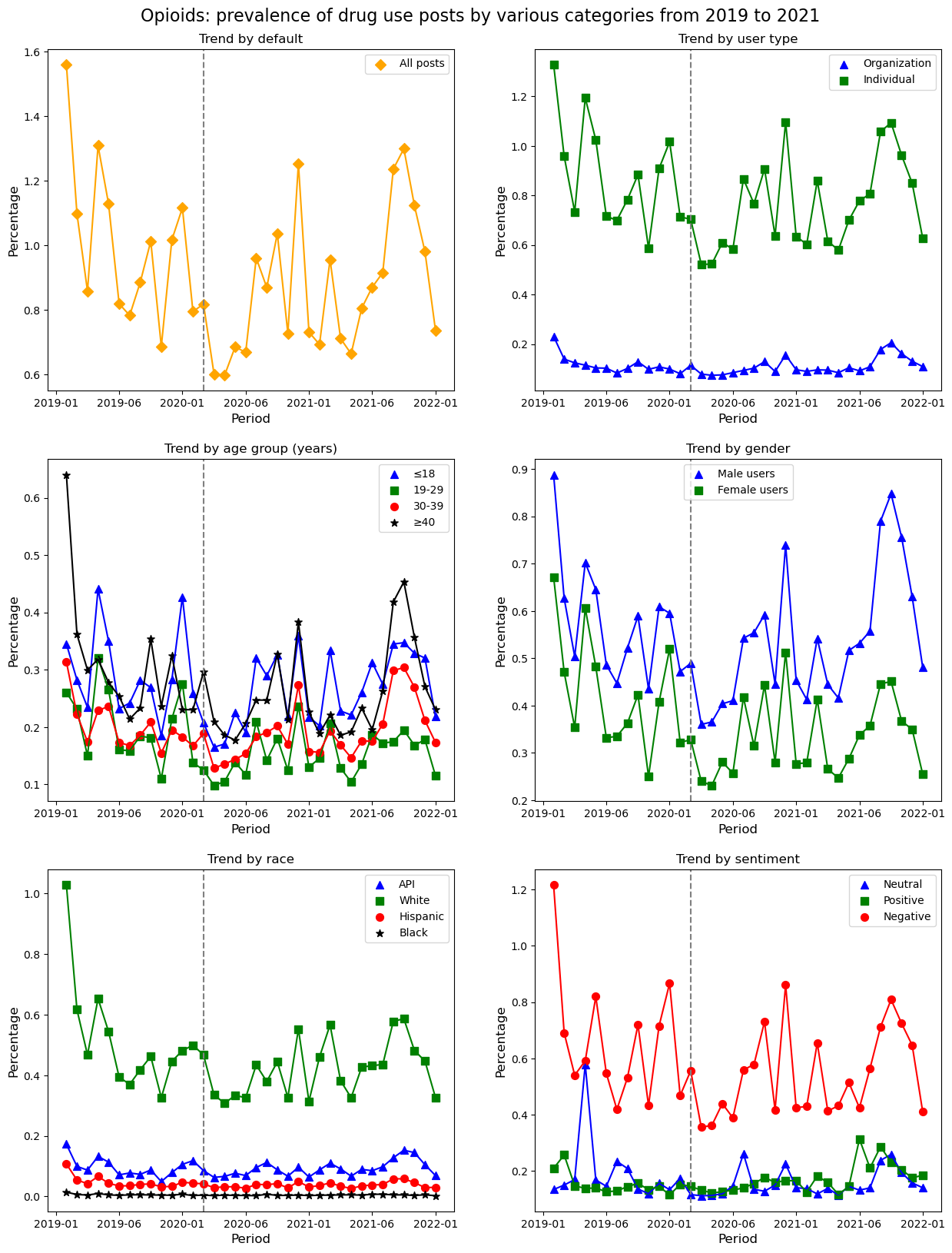


Figure S15. Stimulants posts distribution across six categories from 2019 to 2021
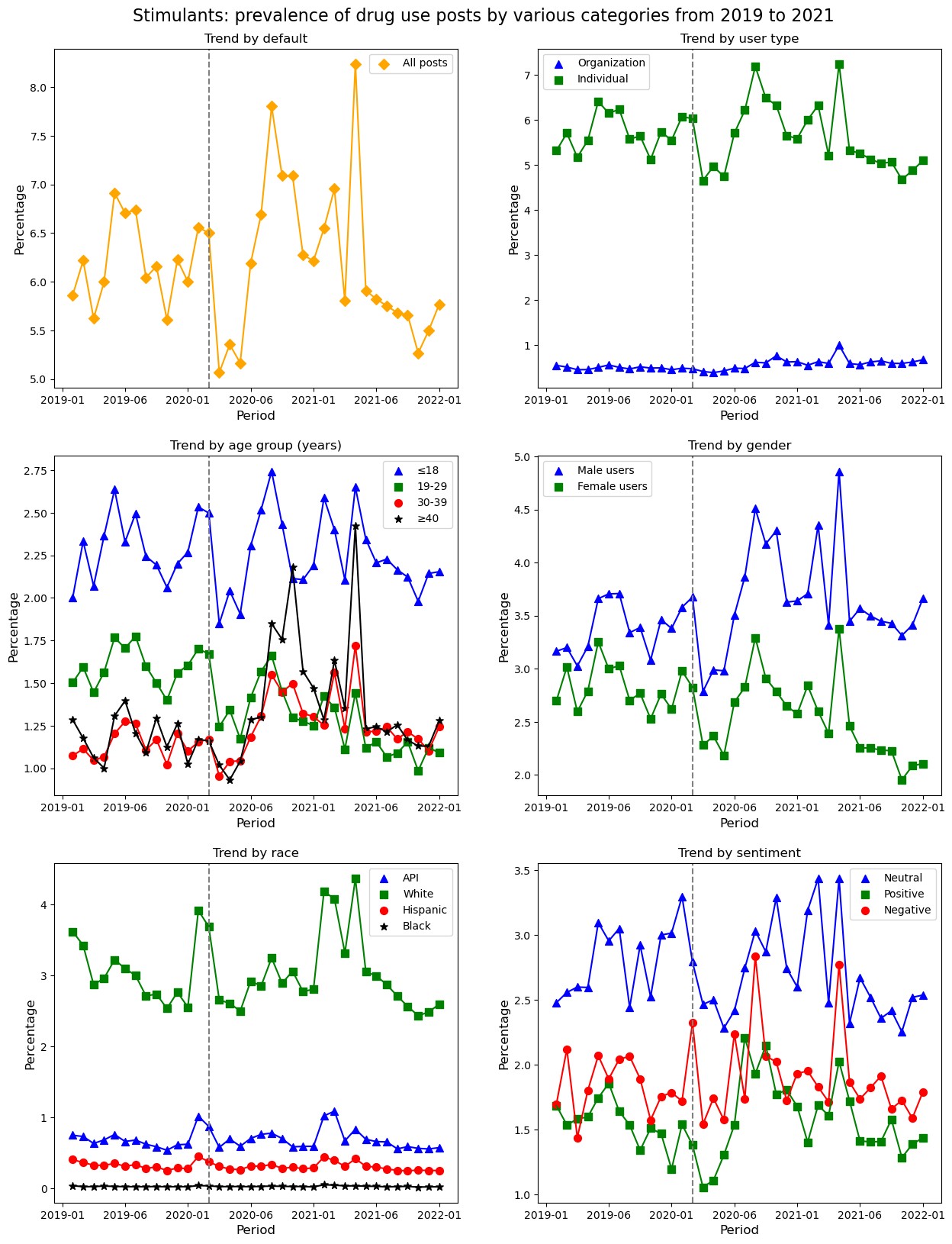


Figure S16. Club drugs posts distribution across six categories from 2019 to 2021


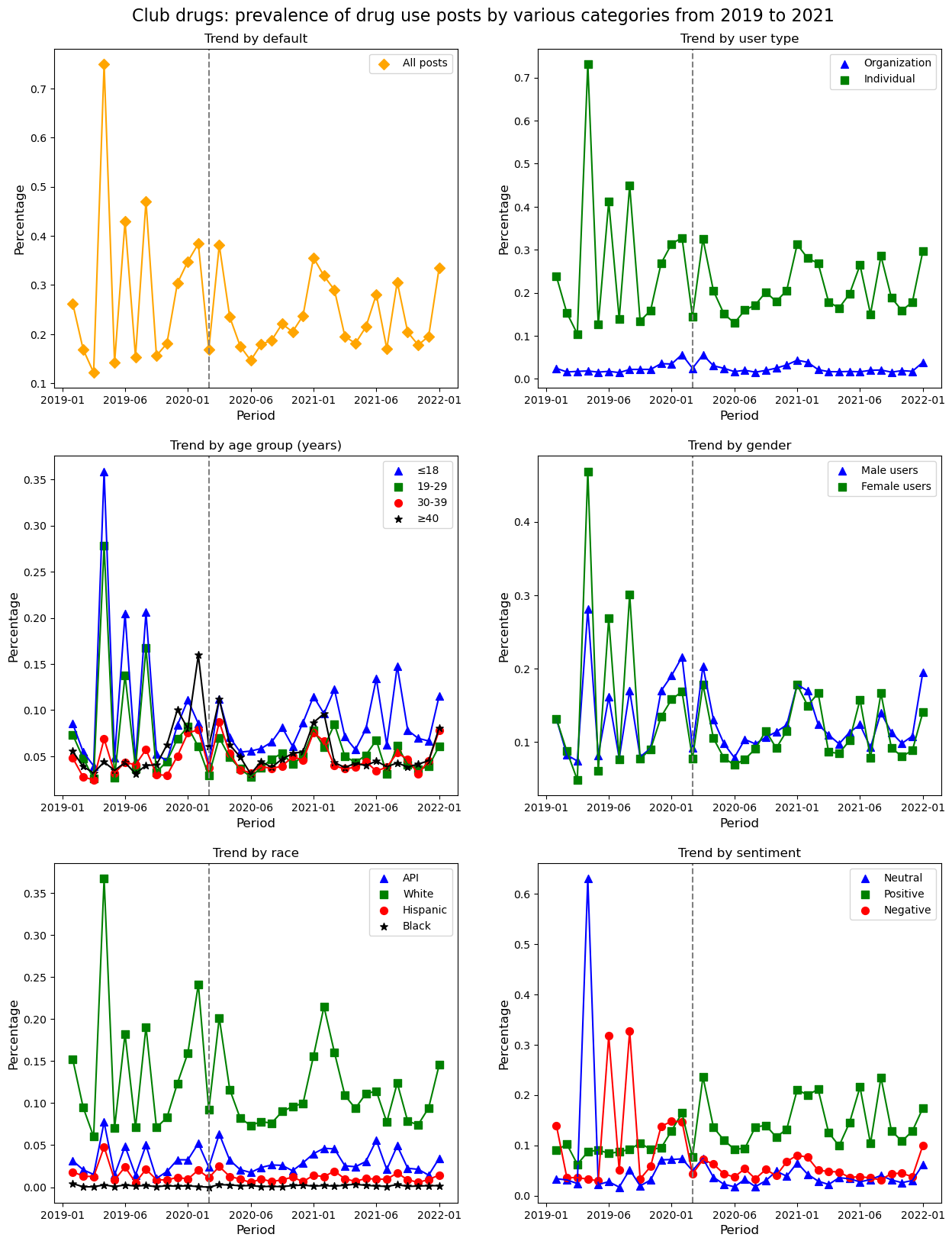


Figure S17. Dissociative drugs post distribution across six categories from 2019 to 2021


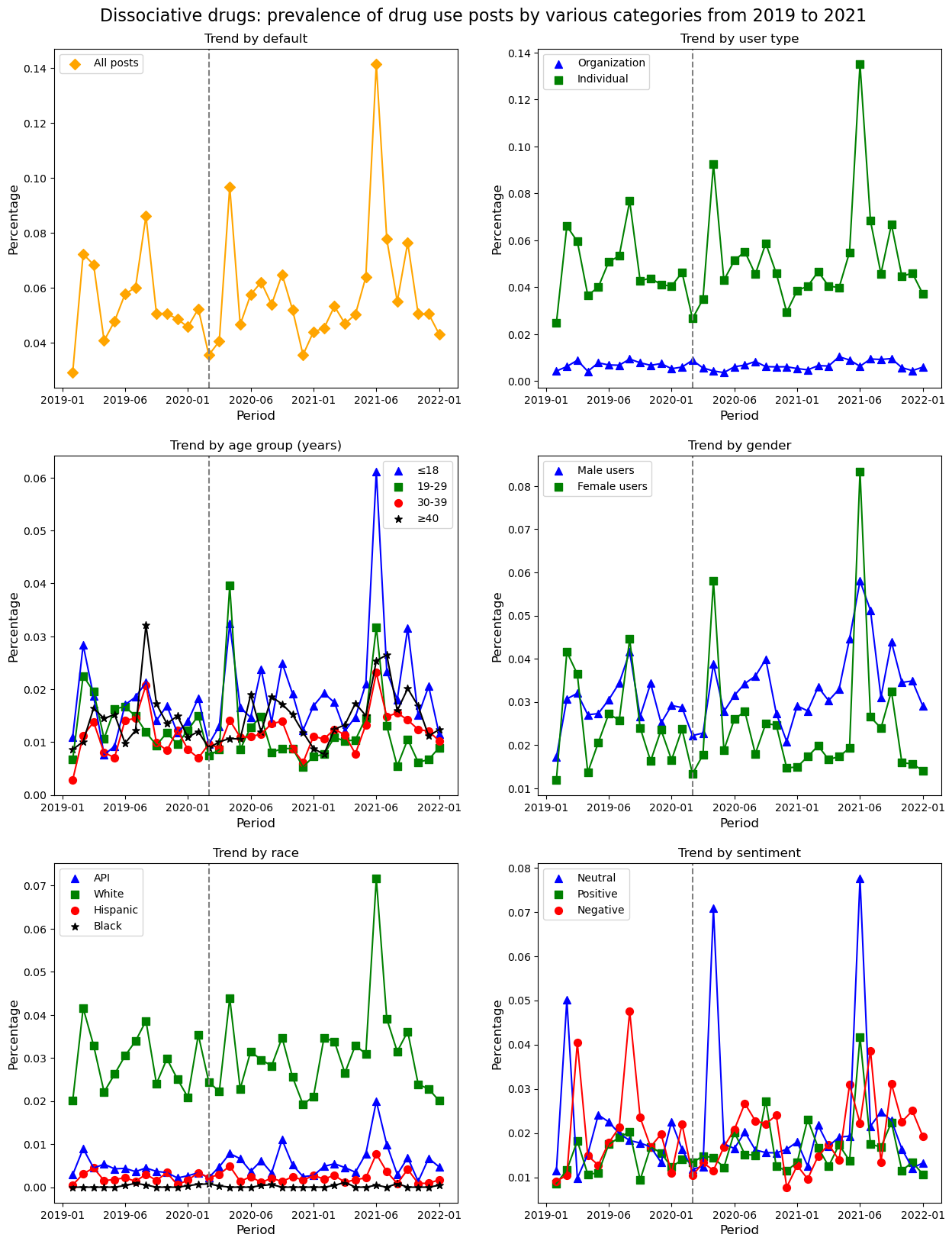


Figure S18. Hallucinogens post distribution across six categories from 2019 to 2021


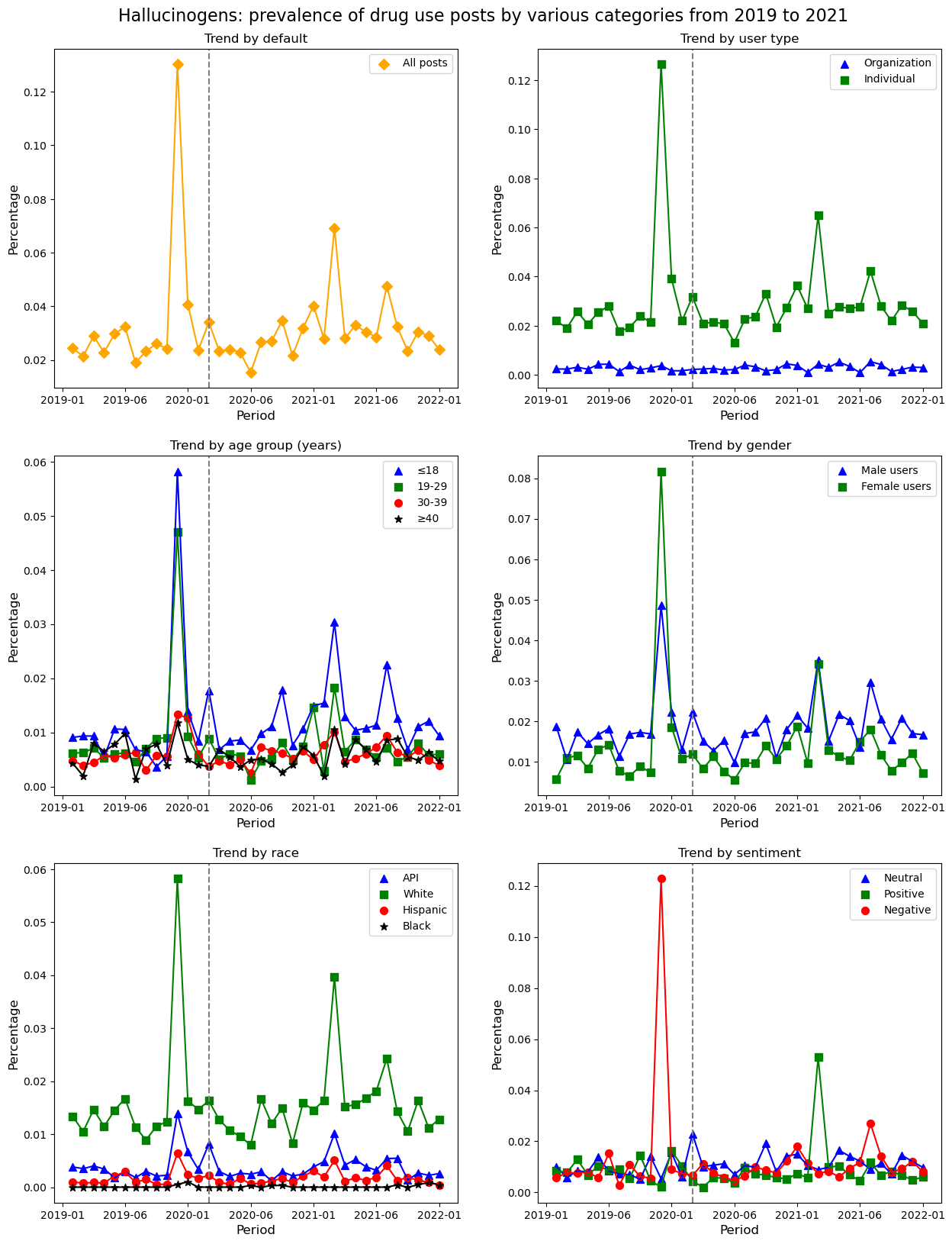


Figure S19. Other compounds post distribution across six categories from 2019 to 2021


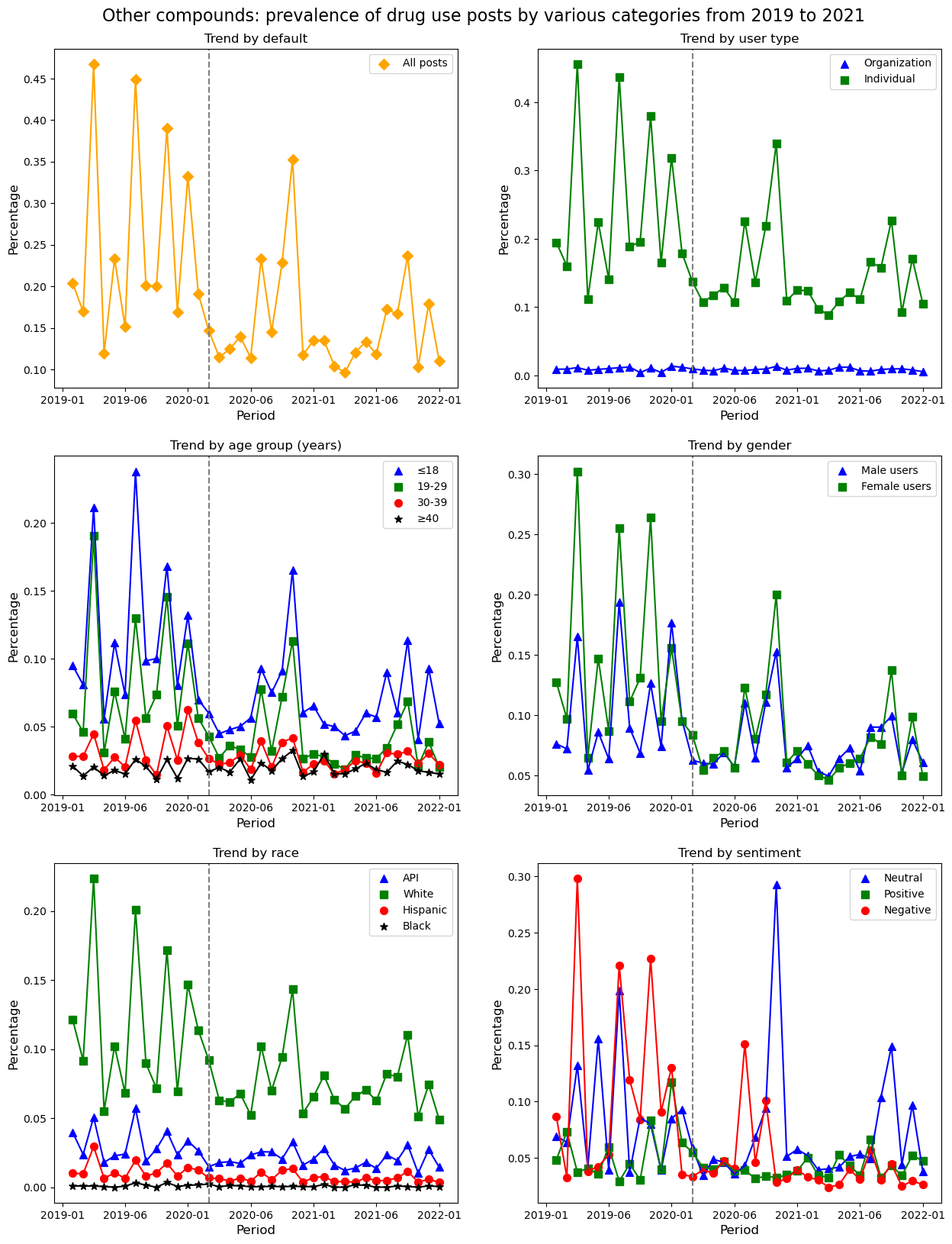


Figure S20. Prescription medications post distribution across six categories from 2019 to 2021
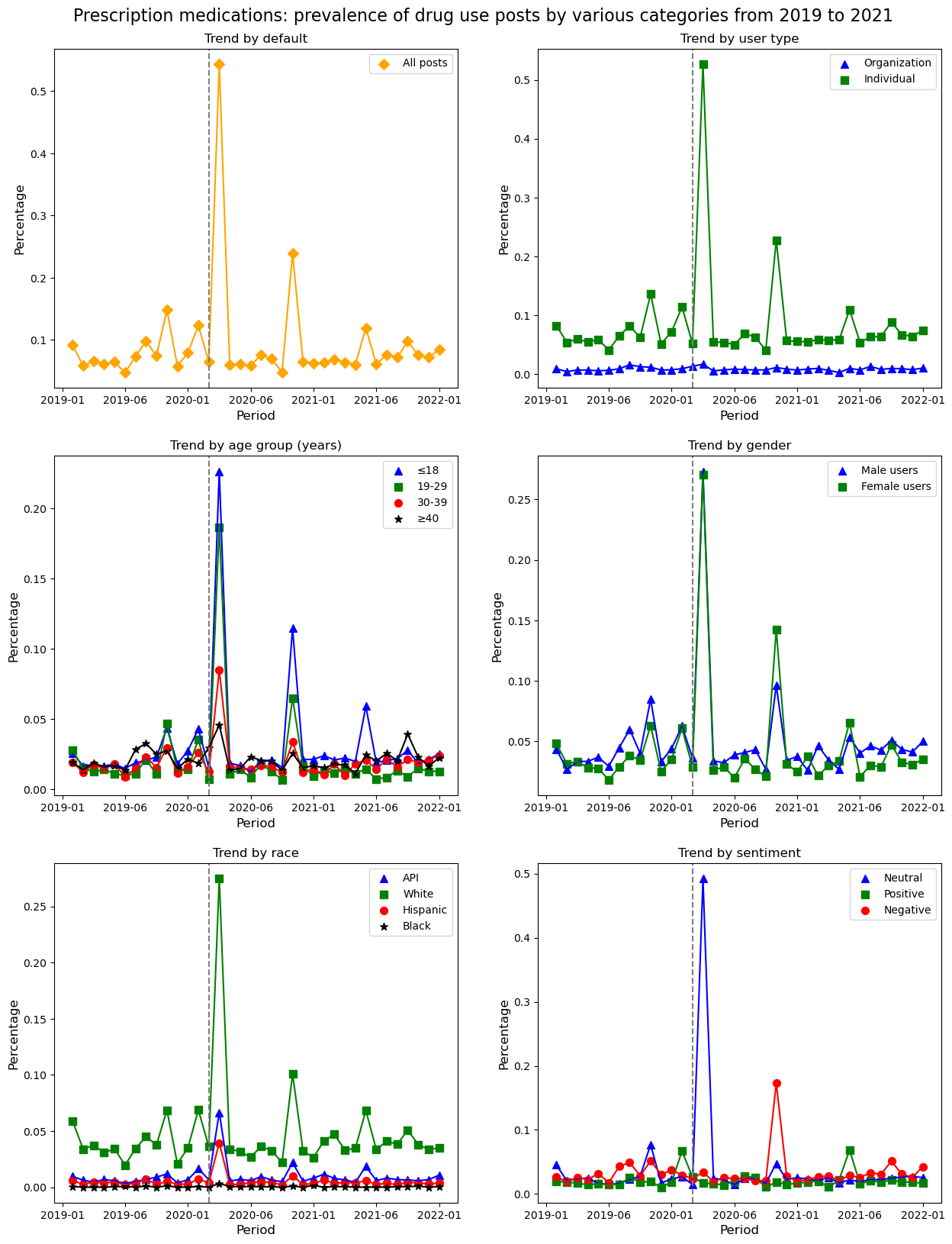

Supplement: Multimedia Appendix 1 [file infodemiology_v5i1e67333_app1.docx]
